# Horizontal Transfer of Bacteriocin Biosynthesis Genes Requires Metabolic Adaptation To Improve Compound Production and Cellular Fitness

**DOI:** 10.1128/spectrum.03176-22

**Published:** 2022-12-06

**Authors:** Sophia Krauss, Theresa A. Harbig, Johanna Rapp, Timm Schaefle, Mirita Franz-Wachtel, Leonie Reetz, Ahmed M. A. Elsherbini, Boris Macek, Stephanie Grond, Hannes Link, Kay Nieselt, Bernhard Krismer, Andreas Peschel, Simon Heilbronner

**Affiliations:** a Department of Infection Biology, Interfaculty Institute of Microbiology and Infection Medicine, University of Tübingen, Tübingen, Germany; b Cluster of Excellence EXC 2124, Controlling Microbes to Fight Infections, Tübingen, Germany; c German Center for Infection Research, Tübingen, Germany; d Interfaculty Institute for Bioinformatics and Medical Informatics, University of Tübingen, Tübingen, Germany; e Department of Bacterial Metabolomics, Interfaculty Institute of Microbiology and Infection Medicine, University of Tübingen, Tübingen, Germany; f Institute of Organic Chemistry, University of Tübingen, Tübingen, Germany; g Proteome Center Tübingen, University of Tübingen, Tübingen, Germany; h Interfaculty Institute of Microbiology and Infection Medicine, Institute for Medical Microbiology and Hygiene, UKT Tübingen, Tübingen, Germany; Lerner Research Institute

**Keywords:** *Staphylococcus aureus*, TCA cycle, bacterial fitness, bacteriocins, metabolic adaptation

## Abstract

Biosynthetic gene clusters (BGCs) encoding the production of bacteriocins are widespread among bacterial isolates and are important genetic determinants of competitive fitness within a given habitat. Staphylococci produce a tremendous diversity of compounds, and the corresponding BGCs are frequently associated with mobile genetic elements, suggesting gain and loss of biosynthetic capacity. Pharmaceutical biology has shown that compound production in heterologous hosts is often challenging, and many BGC recipients initially produce small amounts of compound or show reduced growth rates. To assess whether transfer of BGCs between closely related Staphylococcus aureus strains can be instantly effective or requires elaborate metabolic adaptation, we investigated the intraspecies transfer of a BGC encoding the ribosomally synthesized and posttranslationally modified peptide (RiPP) micrococcin P1 (MP1). We found that acquisition of the BGC by S. aureus RN4220 enabled immediate MP1 production but also imposed a metabolic burden, which was relieved after prolonged cultivation by adaptive mutation. We used a multiomics approach to study this phenomenon and found adaptive evolution to select for strains with increased activity of the tricarboxylic acid cycle (TCA), which enhanced metabolic fitness and levels of compound production. Metabolome analysis revealed increases of central metabolites, including citrate and α-ketoglutarate in the adapted strain, suggesting metabolic adaptation to overcome the BGC-associated growth defects. Our results indicate that BGC acquisition requires genetic and metabolic predispositions, allowing the integration of bacteriocin production into the cellular metabolism. Inappropriate metabolic characteristics of recipients can entail physiological burdens, negatively impacting the competitive fitness of recipients within natural bacterial communities.

**IMPORTANCE** Human microbiomes are critically associated with human health and disease. Importantly, pathogenic bacteria can hide in human-associated communities and can cause disease when the composition of the community becomes unbalanced. Bacteriocin-producing commensals are able to displace pathogens from microbial communities, suggesting that their targeted introduction into human microbiomes might prevent pathogen colonization and infection. However, to develop probiotic approaches, strains are needed that produce high levels of bioactive compounds and retain cellular fitness within mixed bacterial communities. Our work offers insights into the metabolic burdens associated with the production of the bacteriocin micrococcin P1 and highlights evolutionary strategies that increase cellular fitness in the context of production. Metabolic adaptations are most likely broadly relevant for bacteriocin producers and need to be considered for the future development of effective microbiome editing strategies.

## INTRODUCTION

It is increasingly recognized that biosynthetic gene clusters (BGCs) allowing the production of antibacterial compounds are omnipresent in bacterial communities (1). These antibacterial compounds are frequently referred to as bacteriocins. They can be produced either ribosomally or by nonribosomal enzymatic systems and are hugely diverse in terms of molecular size and structure (2). In line with their structural diversity, bacteriocins have diverse molecular targets and killing mechanisms and therefore exhibit toxicity toward diverse spectra of bacterial species (2, 3). Bacteriocin-producing bacterial lineages have recently gained increasing attention for their potential to displace pathogens from various human body sites, thereby preventing infection (4–6).

Gram-positive staphylococci are one example of such bacteria. The genus comprises human commensals that are opportunistic pathogens, such as Staphylococcus epidermidis, Staphylococcus capitis, Staphylococcus lugdunensis, and Staphylococcus haemolyticus, but also the frequently invasive pathogen Staphylococcus aureus. Many staphylococcal isolates show inhibitory activity against a diverse range of human nasal commensals and pathogens (7–9). Interestingly, most staphylococcal BGCs appear to be associated with mobile genetic elements such as plasmids, transposons, insertion sequence (IS) elements, lysogenic phages, and chromosomal islands with G+C contents diverging from the genome average (1, 4, 7, 10). This suggests that the BGCs are transferred between strains and lineages and create strain-specific rather than species-specific antimicrobial properties (1, 11, 12).

Transfer of antibiotic BGCs between strains or species represents a natural system for heterologous expression of antibacterial compounds. Acquisition of such a BGC provides a direct benefit to the recipient, as it allows inhibition of competitors. However, acquisition might also be metabolically challenging for the novel host. Indeed, transfer of BGCs between classical antibiotic-producing bacterial species (e.g., streptomycetes) often results in the production of limited amounts of compound in heterologous hosts, and adaptive mutations or changes in nutritional supplies are needed to optimize compound production (13–16). The same might be true in the context of naturally occurring transfer of BGCs between staphylococcal strains and species. Acquisition of bacteriocin BGCs places a burden on the recipient cell. The novel genetic material needs to be propagated, and, if functionally expressed, it entails production and secretion of large amounts of toxic secondary metabolites. Precursor molecules need to be channeled from primary metabolism, and cellular energy levels might consequently be reduced, entailing metabolic costs for the producer (17). Finally, suboptimal producer immunity against the compound can entail further physiological burdens (18). Accordingly, it seems plausible that BGC acquisition might represent a mixed blessing for bacterial cells. On the one hand, compound production will provide a competitive advantage when susceptible competitors are present. On the other hand, BGC-associated burdens might reduce fitness of the producer and might require adaptive evolution to optimize compound production and fitness. The physiological costs and the mechanisms of integration of bacteriocin synthesis into primary metabolism in staphylococci remain unclear. However, knowledge about this phenomenon is key to understanding why BGCs are largely strain specific and not species-wide conserved traits. Future approaches for the use of bacteriocin-producing bacterial strains to displace pathogens from human microbiomes will crucially depend on the availability of “healthy” strains stably producing high levels of antibiotic molecules.

In this work, we used multiomics approaches to study the transfer of the naturally occurring plasmid pD4-19 between closely related S. aureus strains. This plasmid carries the BGC for the biosynthesis of the thiopeptide bacteriocin micrococcin P1 (MP1). Transfer of pD4-19 to S. aureus RN4220 allowed immediate production of MP1 but caused growth defects. Genome analysis showed that long-term *in vitro* evolution experiments altered the sequence of the citrate synthase-encoding gene to increase translation of this core metabolic enzyme. Metabolome analysis revealed significant changes in the levels of central metabolic molecules, including citrate, α-ketoglutarate, and several amino acids. Transcriptome analysis showed that adaptive evolution also increases the expression of ribosomal proteins as well as of enzymes involved in cofactor biosynthesis and protein turnover, all suggesting increased cellular fitness in the context of augmented MP1 production. Phenotypically, the adaptation enhanced compound production and made it possible to overcome BGC-associated growth defects. Our data indicate that strain-specific genetic and metabolic predispositions will determine the levels of bacteriocin production and fitness of BGC recipients. Hence, this will most likely determine the success of a BGC recipient in the context of competitive environments.

## RESULTS

### MP1 BGCs as a model system.

**FIG 1:**
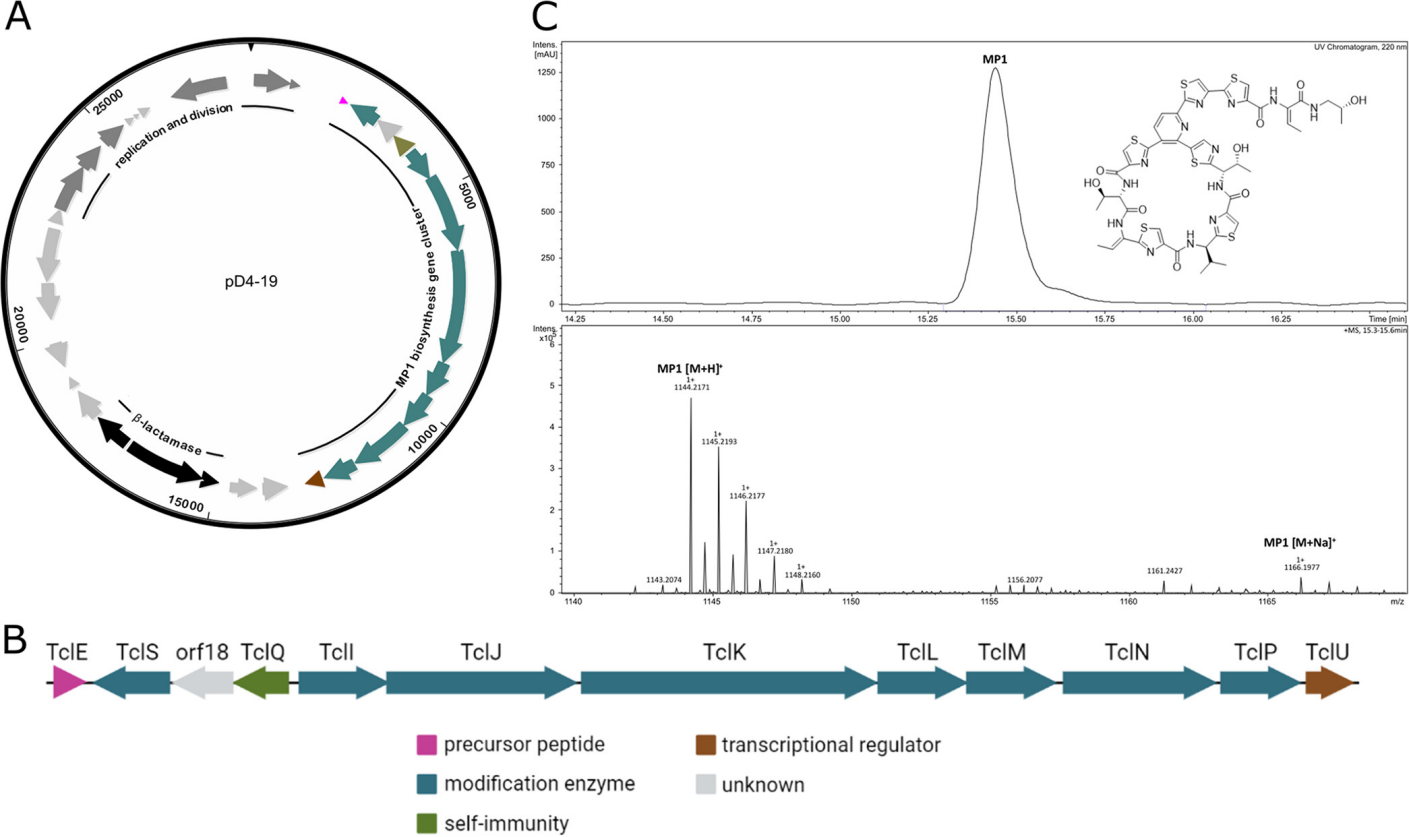
Plasmid pD4-19 carries the BGC for MP1 production. (A) Map of plasmid pD4-19 (28,391 bp), which was identified in S. aureus D4-19 and which carries the MP1 BGC (color), a β-lactamase operon (black), several genes encoding replication and division factors (dark gray), and hypothetical genes (light gray). (B) Detailed view of the MP1 BGC. The annotated protein functions are indicated by different colors. Created with BioRender.com. (C) HPLC-UV chromatogram (220 nm) of methanol extract from the S. aureus D4-19 culture pellet. Absorbance was measured in milli-absorbance units (mAU). The chemical structure of MP1 is depicted. MS spectrum confirming the sum formula of MP1 ([M+H]^+^, *m/z* = 1,144.2171 [found], *m/z* = 1,144.2173 [calculated for C_48_H_50_N_13_O_9_S_6_]).

We sought to investigate the effects of BGC acquisition on the cellular fitness of the recipient cell. Screening of our extensive collection of nearly 1,500 nasal isolates of diverse bacterial species resulted in the identification of two S. aureus strains showing intraspecies inhibition against the test strain, S. aureus USA300 LAC. Whole-genome sequencing (WGS) and antiSMASH (bacterial version 5.0) analysis revealed the presence of plasmids encoding BGCs for aureocin A70 (19) in S. aureus P1-22 and for the thiopeptide MP1 (20) in S. aureus D4-19. The plasmid pD4-19 has a size of 28,391 bp (Fig. 1A) and, besides carrying the 11-kb MP1-encoding BGC (Fig. 1B), encodes a β-lactamase, which we considered useful for *in vitro* plasmid transfer experiments. Therefore, we focused on pD4-19 as a model BGC.

The thiopeptide MP1 is a RiPP (ribosomally synthesized and posttranslationally modified peptide)-type bacteriocin targeting the bacterial ribosome (21), and an alternative ribosomal subunit gene (*tclQ*) provides resistance to the producer (21–23). The MP1 BGC on pD4-19 showed an overall similarity of 99% to the gene cluster identified by Liu et al. in an Staphylococcus hominis isolate (20), suggesting the production of the same compound. To validate this, we performed whole-cell methanol extraction and analyzed the extract by high-performance liquid chromatography coupled with high-resolution electrospray ionization mass spectrometry [HPLC HR ESI positive ion mode (ESI+)-MS]. This analysis revealed the presence of the mass of MP1 (1,144.4 Da with the assigned sum formula of MP1: C_48_H_49_N_13_O_9_S_6_) (Fig. 1C), confirming production of MP1 by S. aureus D4-19. To prove that the identified BGC is responsible for the observed antimicrobial activity, we performed random transposon mutagenesis. Strains without antibiotic activity were found to carry the transposon within the MP1 BGC (see Fig. S1 in the supplemental material), confirming that MP1 alone was responsible for the antibiotic activity of S. aureus D4-19.

### Acquisition of the MP1 BGC enables MP1 production but imposes a metabolic burden.

**FIG 2:**
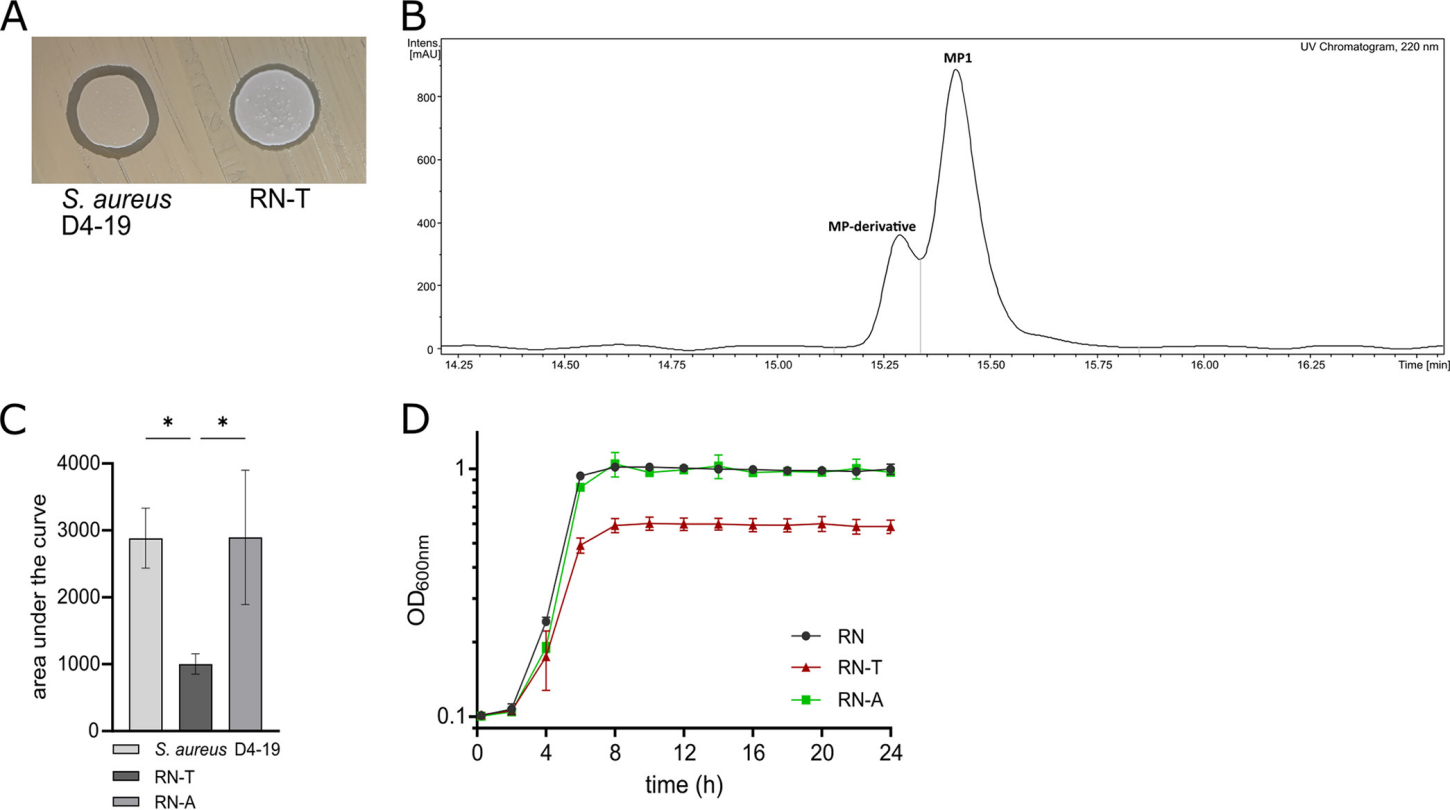
Acquisition of BGC allows production of MP1 but imposes a fitness cost, which can be overcome through adaptive evolution. (A) Spot assay of the nasal isolate S. aureus D4-19 and the S. aureus recipient strain RN-T on S. aureus USA300 LAC, demonstrating antimicrobial activity of both strains against the indicator strain. (B) HPLC-UV chromatogram (220 nm) of cell pellet extracts of RN-T, confirming the production of MP1. An MP derivative (exact mass *m/z* = 1,163.2116), with a retention time of 15.3 min, is also produced by this strain. (C) Estimation of the relative quantity of MP1 produced by S. aureus strains D4-19, RN-T, and RN-A. MP1 amount is estimated as curve integral (*n* = 3). The adapted strain RN-A produces amounts similar to those of the nasal isolate. MP1 production is significantly reduced in the newly transformed strain RN-T. (D) Growth curves of S. aureus RN, RN-T, and RN-A grown in BM over 24 h in a 24-well plate (*n* = 4). Statistical significance was determined using an ordinary one-way analysis of variance (ANOVA) (Tukey’s multiple-comparison test) (*, *P* < 0.05).

We sought to investigate whether pD4-19 can be transferred to a related S. aureus strain and whether this entails significant physiological changes. The plasmid isolated from S. aureus D4-19 was used to transform S. aureus RN4220 by electroporation. A recovered transformant (RN-T) showed antimicrobial activity against S. aureus USA300 LAC, suggesting the production of MP1 (Fig. 2A). However, the zone of inhibition was smaller than that of the original producer S. aureus D4-19. HPLC-MS and nuclear magnetic resonance (NMR) analysis of cell extracts showed that RN-T produced MP1 as well as a putative derivative with a mass of 1,163.2116 Da (Fig. 2B; Fig. S2 to S4). However, the combined amount of MP1 and the putative derivative was 3-fold lower than the amount of MP1 produced by S. aureus D4-19 (Fig. 2C).

Growth curve analysis showed that S. aureus RN-T reached lower optical densities than the parental strain, S. aureus RN4220 (RN) (Fig. 2D), suggesting that MP1 production may limit the proliferation of the strain. To verify that the reduced growth was caused by MP1 production and not by other plasmid-associated effects, a mutant lacking the entire MP1 operon (*tclESQIJKLMNPU* plus orf18) was constructed by allelic replacement. The resulting strain, S. aureus pD4-19 ΔMP1, lacked antimicrobial activity against S. aureus USA300 LAC (Fig. S5B) and reached an optical density at 600 nm (OD_600_) similar to that of S. aureus RN4220 (Fig. S5A). This supports the idea that acquisition of the MP1 BGC and the associated MP1 production impose a physiological burden on S. aureus RN4220. It has been reported that insufficient immunity to an antimicrobial product can limit production of the compound and growth of the producer (18). However, S. aureus D4-19 and RN-T mutant strains lacking the MP1 structural gene *tclE* and therefore deficient in MP1 production were highly resistant to purified MP1 (MIC > 100 μg/mL). This finding indicates that the immunity to MP1 conferred by the alternative ribosomal subunit TclQ is strong enough in both strain backgrounds to allow effective growth even during high-level MP1 production (Fig. S5C).

### Adaptive evolution increases MP1 production and relieves the metabolic burden.

We speculated that MP1 biosynthesis might perturb the primary metabolism of S. aureus RN4220, thereby limiting bacterial growth and propagation. To analyze whether RN4220 can adapt its metabolism to MP1 production, RN-T was passaged daily in basic medium (BM) over 28 consecutive cultures. Colonies arising on solid medium at the end of the experiment were found to be larger than those formed by the original transformant, suggesting improved growth. This adapted strain was named RN-A (S. aureus RN4220 pD4-19 adapted). Growth curve analysis confirmed that the new strain reached OD_600_ values similar to those of S. aureus RN (Fig. 2D). Additionally, S. aureus RN-A produced levels of MP1 comparable to those of the native MP1 producer S. aureus D4-19 and 2.86-fold greater than those of the nonadapted parental strain, RN-T (Fig. 2C).

**FIG 3:**
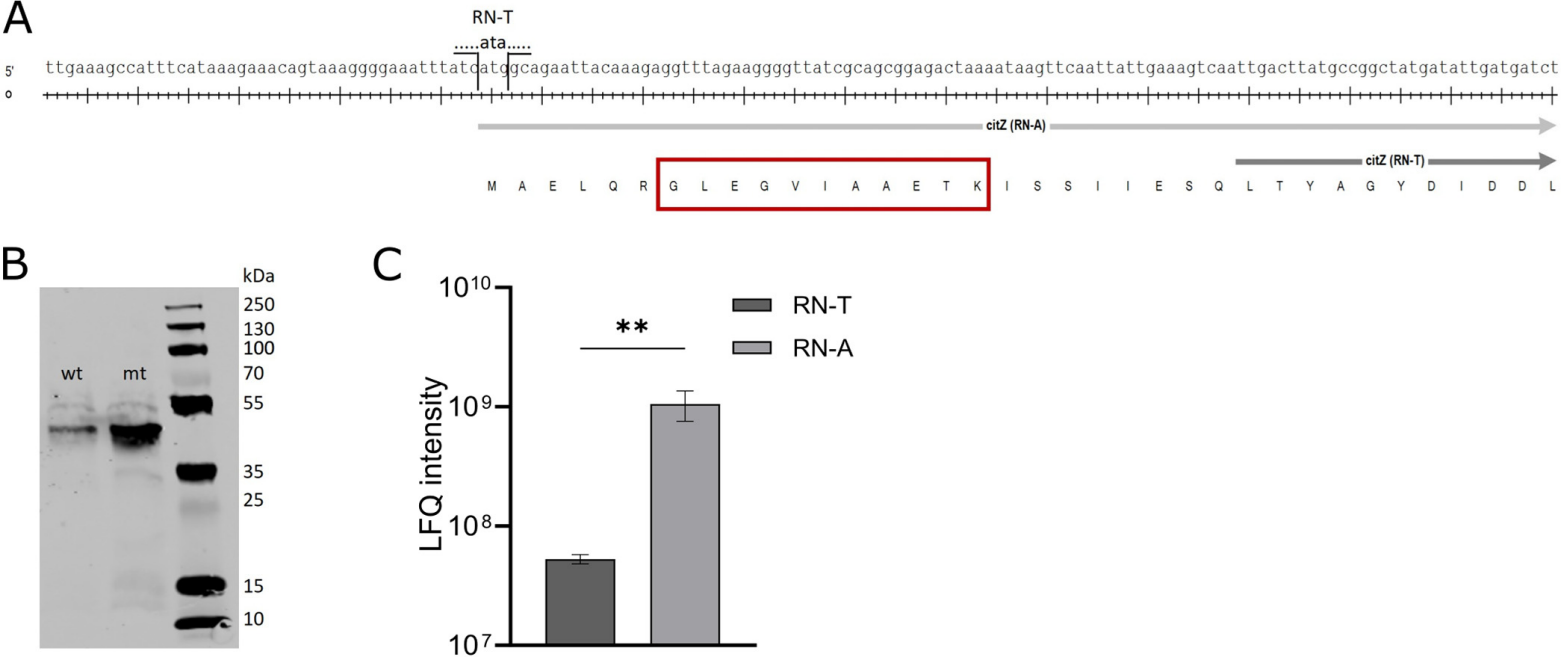
An adaptive mutation in the *citZ* gene increases the levels of the citrate synthase CitZ. (A) Promoter region and start site of *citZ* (ACCFDFCE_01589) gene from RN-T (WT) (dark gray) and RN-A (light gray). The WT *citZ* has a delayed start codon compared to the RN-A allele, where ATA is changed to ATG, leading to an earlier transcription start of *citZ* in RN-A. This mutation reverts CitZ to a full-size protein, similar to that of other staphylococci. The red box highlights the part of the amino acid sequence of CitZ that was used for the analysis shown in panel C. (B) Western blot of cell extracts from S. aureus RN4220 pRB473-XylR-6xHis-*citZ* (wt) and S. aureus RN4220 pRB473-XylR-6xHis-*citZ* (mt) expressing the WT and the mutant CitZ proteins, respectively. CitZ has a size of 42.6 kDa and can be detected in both extracts, but in larger amounts in the mutant-CitZ-expressing strain, indicating leaky expression of WT CitZ. (C) Proteome data were analyzed for the presence of the protein fragment GLEGVIAAETK (depicted in panel A [*n* = 3]) to confirm leaky expression of WT CitZ with ATA as the start codon. The indicated fragment can be found in both proteomes, but with a 20-fold increase in RN-A. Statistical significance was determined using an unpaired *t* test (**, *P* < 0.01).

To identify mutations explaining the phenotypic differences between RN-T and RN-A, the strains were subject to whole-genome sequencing, and single nucleotide polymorphisms (SNPs) were extracted. We identified a SNP in RN-A, which was located upstream of the gene *citZ* (RN4220 ACCFDFCE_01589), encoding citrate synthase. The mutation created a functional in-frame start codon (ATG instead of ATA), extending the annotated open reading frame of *citZ* by 75 nucleotides (Fig. 3A). An AG-rich motif resembling a Shine-Dalgarno sequence is present 6 bp upstream of the new start codon, creating a canonical translational start site. In contrast, the annotated shorter allele of S. aureus RN4220 lacks an obvious Shine-Dalgarno sequence and relies on a noncanonical TTG start codon, which is rarely used in S. aureus (24). Accordingly, we speculated that the *citZ* gene of S. aureus RN4220 is truncated and potentially nonfunctional. When the *citZ* allele of S. aureus RN4220 was compared with those of other S. aureus strains, the shorter *citZ* allele was found in the entire clonal lineage of S. aureus RN4220, including the ancestral strains NRS146, NRS133, and VC40, derived from S. aureus NCTC8325, which was originally described in 1965 (25). The other 3,834 S. aureus genome sequences of the NCBI database, including the original host of the plasmid, S. aureus D4-19, carry the full-length *citZ* allele found in the adapted strain, RN-A.

Accordingly, we hypothesized that RN4220 possesses a malfunctioning *citZ* allele whose functionality is restored by the adaptive mutation. To test this, the full-length *citZ* gene of the adapted strain plus its Shine-Dalgarno sequence as well as the corresponding sequence of RN4220 were cloned with a C-terminal His tag in the S. aureus vector pRB473-XylR, enabling xylose-inducible expression in RN4220. Upon induction, protein levels were assessed by infrared Western blotting. Interestingly, we found that expression of both alleles resulted in the production of apparently full-length CitZ proteins (43 kDa) (Fig. 3B). This suggested that the malfunctioning allele is translated using the ATA codon as a noncanonical start codon to produce full-length CitZ, a phenomenon that has been observed in Escherichia coli (26). However, the ATG mutation of the same codon in the adapted allele increased CitZ levels dramatically (Fig. 3B), supporting the idea that the adaptive mutation increases the cellular levels of CitZ by creating an appropriate translational start. This hypothesis was further confirmed by subsequent proteome analysis. An 11-amino-acid motif (GLEGVIAAETK) that can result only from the fragmentation of full-length CitZ was found in both strains. However, this fragment was 20-fold more abundant in RN-A than in RN-T (Fig. 3C). This confirmed that both strains translate the CitZ mRNA using the ATA/ATG codon affected by the point mutation. However, the efficiency of translation is strongly increased by the adaptive mutation, resulting in increased levels of CitZ.

### Increased citrate levels make it possible to overcome the MP1-associated burden.

**FIG 4:**
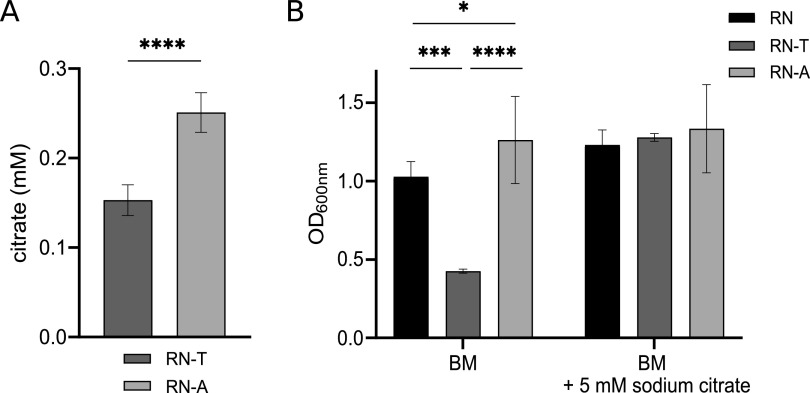
Increased levels of citrate can restore wild-type levels of growth in the MP1 BGC recipient strain RN-T. (A) Significantly reduced citrate levels in RN-T compared to RN-A were determined using the citrate assay kit from Sigma-Aldrich (*n* = 3). Statistical significance was calculated using an unpaired *t* test (****, *P* < 0.0001). (B) Endpoint OD_600_ of S. aureus RN, RN-T, and RN-A in standard growth medium or medium supplemented with 5 mM sodium citrate. Strains were grown in a 24-well plate for 24 h (*n* = 3). Statistical significance was calculated using an ordinary one-way ANOVA (Tukey’s multiple-comparison test) (*, *P* < 0.05; ***, *P* < 0.001; ****, *P* < 0.0001).

To investigate the effects of the mutation on the cellular metabolism, we measured the intracellular concentration of citrate using a colorimetric assay. We found increased levels in RN-A compared to RN-T (Fig. 4A), reflecting the increased amounts of CitZ upon adaptation. Notably, addition of 5 mM sodium citrate to the culture medium allowed the original transformant, RN-T, to overcome the bacteriocin-associated growth defects (Fig. 4B), indicating that intracellular citrate levels represent the limiting factor for growth of MP1-producing S. aureus RN4220.

### The adaptive mutation increases the metabolic activity of the MP1 producer.

**FIG 5:**
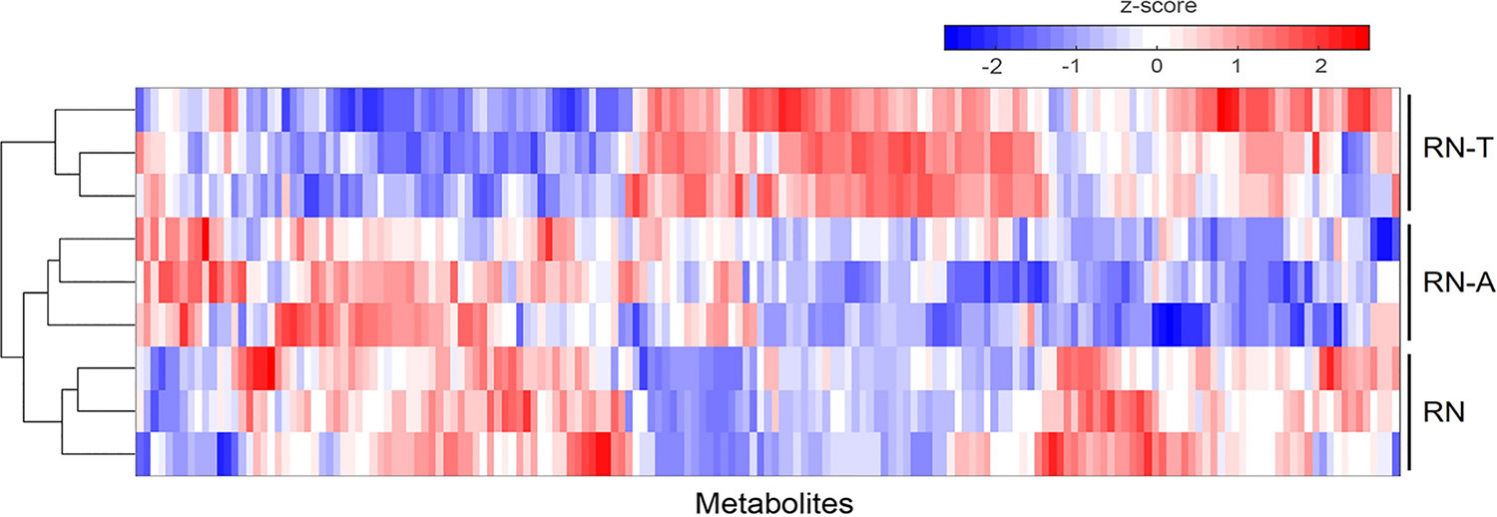
Acquisition of the MP1 BGC alters the metabolite profile of the recipient strain RN-T, an effect that can be reverted by the adaptive mutation. The heat map shows data from FI-MS analysis for the indicated strains. Depicted are z-score-normalized intensities of *m/z* features in positive and negative ionization mode that were annotated to metabolites (means for 3 replicates). Samples were grouped by hierarchical clustering, as indicated by the dendrograms.

To gain insights into the overall levels of primary metabolites, we performed flow injection mass spectrometry (FI-MS)-based untargeted metabolomics of cell lysates of S. aureus RN, the initial transformant (RN-T), and the adapted strain (RN-A). Hierarchical clustering showed that the metabolic profiles of RN and of RN-A were more similar to each other than to those of RN-T (Fig. 5). This finding indicated that the acquisition of the BGC-carrying plasmid impacts the general metabolism of the recipient, and these effects are largely corrected by the adaptive mutation of *citZ*.

**FIG 6:**
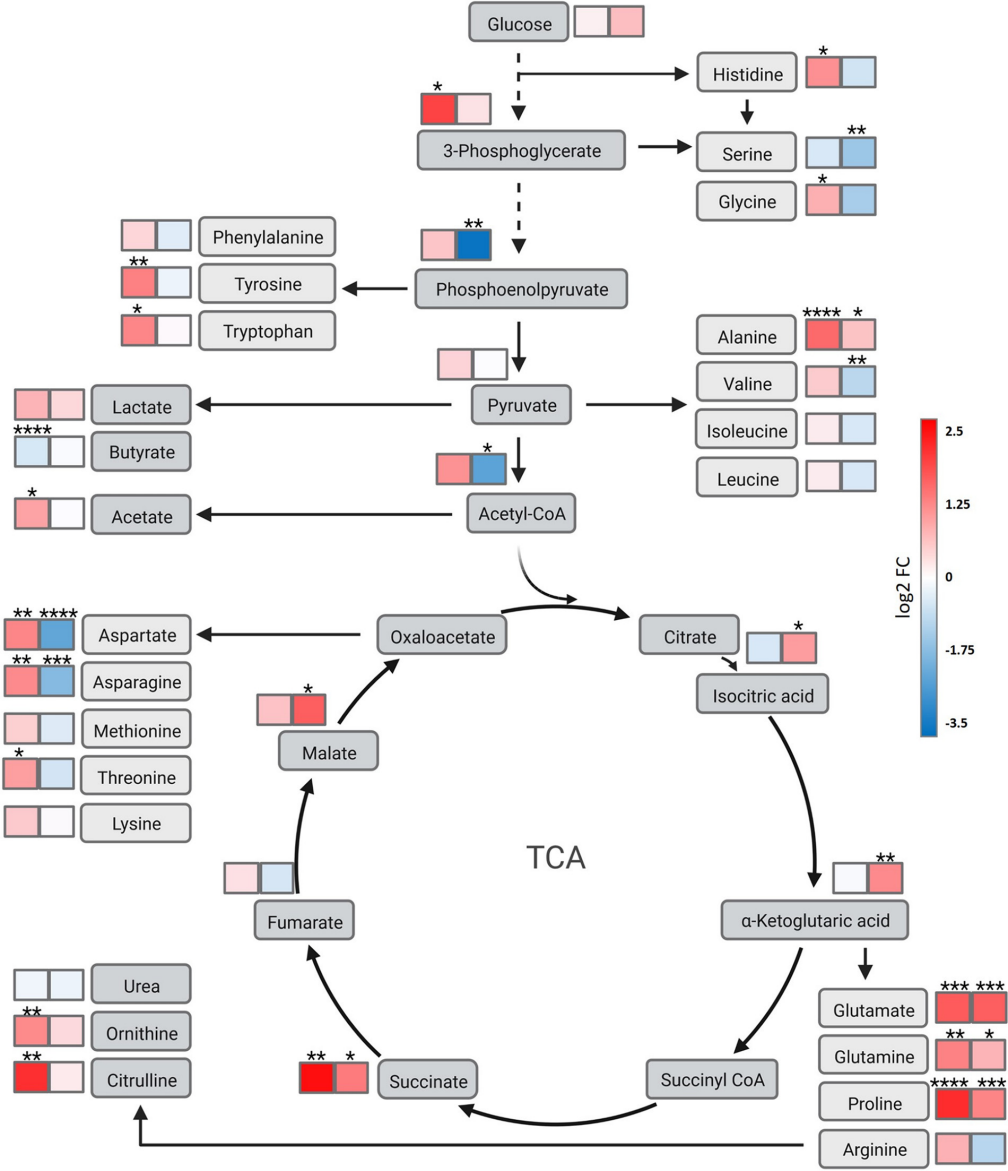
Overview of the metabolic differences between the recipient strain RN-T and the adapted strain. Metabolic differences of RN-T (left boxes) and RN-A (right boxes) are shown as log_2_ fold changes relative to S. aureus RN4220. Red shows an increase in metabolite levels compared to S. aureus RN4220. Blue shows a decrease of metabolite levels compared to S. aureus RN4220. Metabolic pathways are not depicted in detail. Metabolites were measured via FI-MS and LC-MS/MS. The *P* values were calculated using an ordinary one-way ANOVA (Tukey’s multiple-comparison test). *, *P* < 0.05; **, *P* < 0.01; ***, *P* < 0.001; ****, *P* < 0.0001. Created with BioRender.com.

RiPP-type bacteriocins rely on the availability of appropriate amino acids as well on availability of ATP. Precursors for the biosynthesis of amino acids are derived from intermediates of glycolysis, the pentose phosphate pathway and tricarboxylic acid (TCA) cycle. Therefore, we used a combination of liquid chromatography-tandem mass spectrometry (LC-MS/MS) and FI-MS analysis to investigate the levels of the central metabolites with the highest possible accuracy (Fig. 6; Fig. S6 to S9). Relative to the wild type (WT), the levels of glycolysis intermediates (3-phosphoglycerate, phosphoenolpyruvate, pyruvate, and acetyl coenzyme A [acetyl-CoA]) increased upon plasmid acquisition and decreased to or even below RN levels upon adaptive mutation (Fig. 6; Fig. S7B). This suggests that the TCA cycle is insufficiently fed upon plasmid acquisition and that the adaptive mutation restores efficient feeding. In line with this, we found increased levels of citrate (citrate and its isobars, isocitrate and 5-dehydro-4-deoxy-d-glucarate, cannot be discriminated in this analysis) as well as of α-ketoglutarate upon adaptive mutation (Fig. 6; Fig. S7A), supporting the stimulative effect.

Interestingly, we found that pD4-19 acquisition led to the accumulation of various amino acids (aspartate and asparagine, alanine, tyrosine and tryptophan, threonine, glycine, and histidine), pointing to a reduced rate of protein biosynthesis (27). Amino acids did not accumulate in the CitZ-adapted strain. In contrast, levels of most amino acids were reduced in RN-A compared to those in RN, suggesting their efficient usage in protein biosynthesis (Fig. 6; Fig. S6). Exceptions were glutamate, glutamine, and proline, which increased in both RN-T and RN-A relative to RN. Glutamate and glutamine serve as precursors for several amino acids (including proline), and bacteria form glutamate or glutamine by condensation of α-ketoglutaric acid or glutamate with ammonium as a means to acquire environmental nitrogen for anabolic processes. Accordingly, their levels are regarded as an indicator of nitrogen availability (28), and they connect the urea cycle to amino acid biosynthesis. Accumulation of urea cycle intermediates (ornithine, citrulline, and argininosuccinate) was observed in RN-T but not upon adaptive mutation, suggesting that the intermediates are efficiently used to feed the TCA cycle and, with it, amino acid biosynthesis in the adapted strain (Fig. 6; Fig. S8).

**FIG 7:**
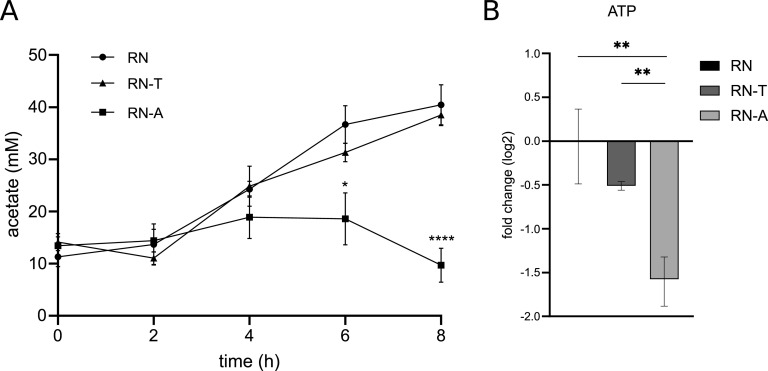
The increased intracellular levels of acetate in RN-T cannot be ascribed to reduced levels of ATP. (A) Extracellular acetate levels were measured in S. aureus RN, RN-T, and RN-A after 0, 2, 4, 6, and 8 h of growth in BM with the acetate assay kit from Sigma-Aldrich (*n* = 3). (B) Changes (log_2_ fold) of cytoplasmic ATP levels in S. aureus RN-T (dark gray) and RN-A (light gray) compared to RN (black) (*n* = 3). ATP was measured via LC-MS/MS. The *P* values were calculated using an ordinary one-way ANOVA (Tukey’s multiple-comparison test). *, *P* < 0.05; **, *P* < 0.01; ****, *P* < 0.0001.

Besides the provision of precursors for metabolic processes, the activity of the TCA cycle is crucial for the aerobic generation of ATP. In the presence of glucose, S. aureus produces ATP by substrate-level phosphorylation even in the presence of oxygen and produces predominantly acetate, which is secreted. Only after glucose is depleted is acetate consumed and decarboxylated using the TCA cycle and the respiratory chain (29, 30). This metabolic switch has been reported to occur after approximately 5 h of growth in glucose-containing complex medium (30), which matches the observed starting point of growth deficiency in our experiments. We noted that the intracellular levels of acetate were increased in RN-T compared to RN (Fig. 6; Fig. S9). We therefore speculated that CitZ and TCA cycle activity might not allow sufficient ATP generation when acetate needs to be catabolized. Interestingly, we detected accumulation of acetate in the culture supernatants of S. aureus RN4220 and of RN-T, while accumulation was strongly reduced in RN-A and dropped quickly after 6 h of growth (Fig. 7A). This suggests an increased rate of acetate consumption and oxidative decarboxylation upon restoration of the *citZ* allele. However, ATP levels measured by metabolome analysis did not support energy depletion as the reason for the growth defect of RN-T (Fig. 7B). Compared to the WT strain, ATP levels were only moderately decreased in RN-T, and the decrease intensified upon adaptive mutation. As RN-A does not show abnormal growth, these data suggest that even the lowest ATP levels observed in RN-A are sufficient to support maximal growth.

### Transcriptomic signatures support increased overall metabolic activity upon adaptive mutation.

**FIG 8:**
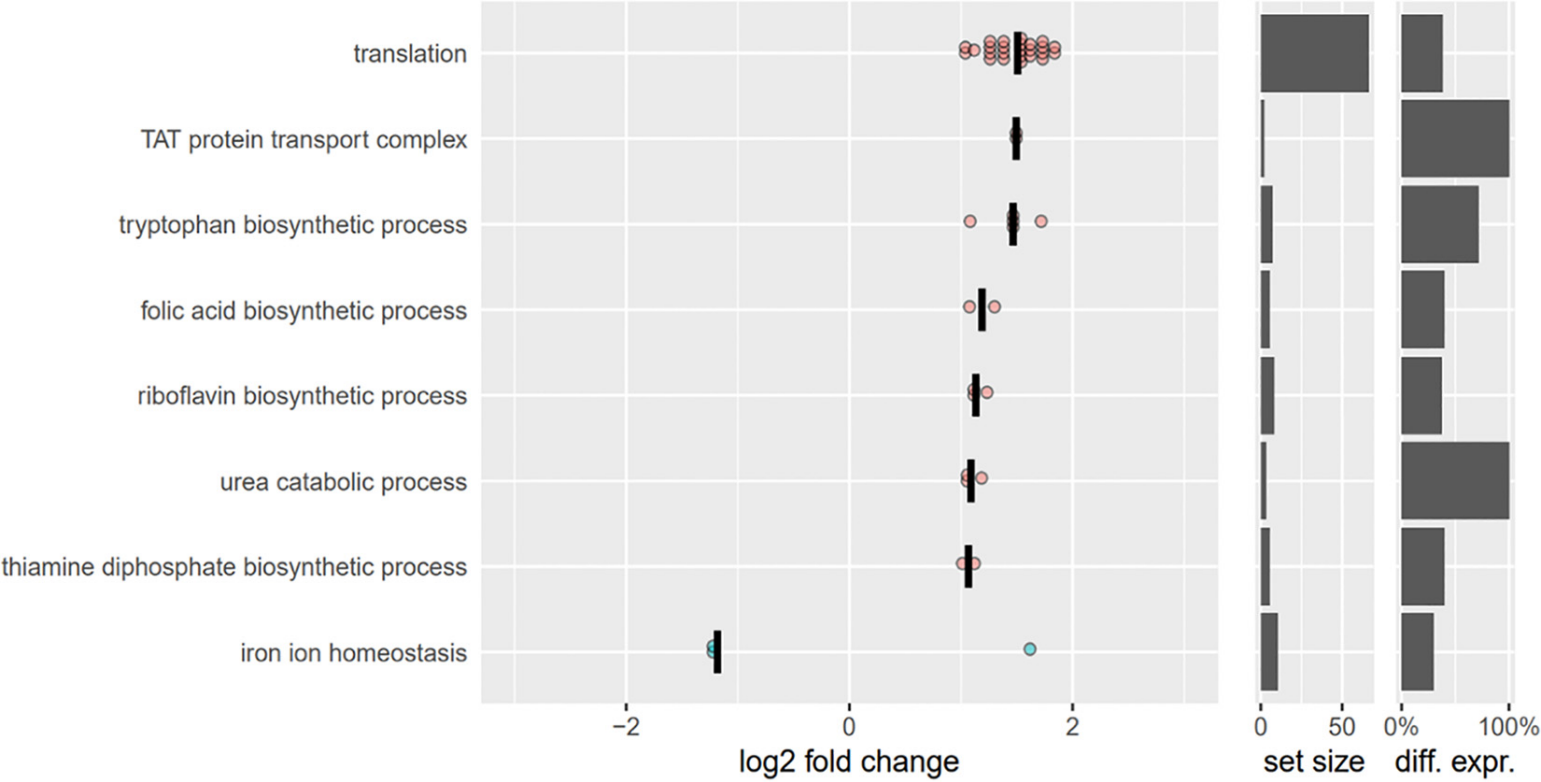
The adaptive mutation increases the overall metabolic activity of strain RN-A, as confirmed by transcriptomic analysis. Selection of significantly enriched GO terms and the corresponding differentially expressed genes obtained via RNA-seq. Data are the log_2_ fold change in expression levels of S. aureus RN-A compared to RN-T. Increased median expression is indicated in red; decreased median expression is in blue. The set size indicates the number of genes covered by the GO term. The last column indicates how many genes of the set are differentially expressed (percent).

We performed transcriptome sequencing (RNA-Seq) analysis of RN-T and RN-A to investigate the effects of the adaptive mutation on the transcriptome. We extracted differentially regulated genes and assigned them to Gene Ontology (GO) groups to identify the general cellular functions altered in response to the adaptive mutation in *citZ*. The adaptive mutation entailed increased transcription of the translational machinery (30S and 50S ribosomal subunits), pointing to generally enhanced protein biosynthesis as a consequence of overall metabolic alterations (Fig. 8). This change was accompanied by upregulation of pathways for the biosynthesis of the cofactors folate (*folPBK*), thiamine (*thiEM*), and riboflavin (*ribAB*). Increased amino acid turnover was reflected by upregulation of the l-tryptophan biosynthesis pathway (*trpCDEFG*) as well as of catabolic pathways for threonine (*ilvA*) and alanine (*ald1*). Additionally, upregulation of the genes encoding the urea transporter Utp and of each gene of the urease operon (*ureABCEFGD*), catalyzing the hydrolysis of urea into carbon dioxide and ammonia, was observed (Fig. 8; Fig. S10A). Furthermore, upon plasmid acquisition, several genes associated with iron homeostasis as well as the genes encoding the twin-arginine translocation (Tat) pathway were differently expressed (Fig. 8). The Tat system is responsible for the translocation of the iron-dependent peroxidase (FepB), which is also involved in iron uptake (31). Accordingly, one can speculate that the increased metabolism upon adaptive mutation also influences iron homeostasis. Reasons for this are unclear, but TCA-derived citrate is the central molecule needed for production of the S. aureus siderophore staphyloferrin A (32). It seems likely that a secondary effect of the adaptive mutation is optimization of siderophore production, resulting in increased cellular iron levels.

Of note, expression of plasmid associated genes did not differ significantly between RN-T and RN-A (Fig. S10B).

**FIG 9:**
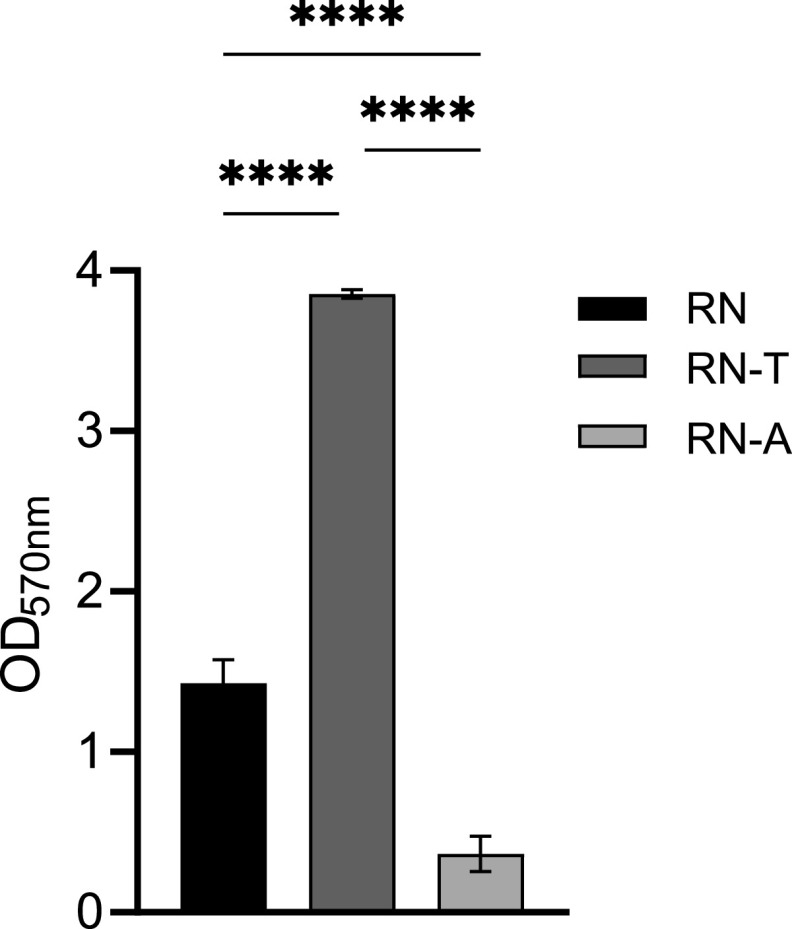
Acquisition of the MP1 BGC is associated with increased levels of biofilm formation. Biofilm formation of S. aureus RN, RN-T, and RN-A grown in BM (*n* = 3). Statistical significance was calculated using an ordinary one-way ANOVA (Tukey’s multiple-comparison test) (****, *P* < 0.0001).

Inclusion of the transcriptomic profile of S. aureus RN in this analysis allowed us to identify several additional effects of plasmid acquisition on the transcriptome. We found transcription of the carnitine transporter *opuC* (*opuCA*, -*CB*, -*CC*, -*CD*) to be downregulated in S. aureus RN-T compared to RN. This is in line with reduced carnitine levels detected via metabolome analysis (Fig. S9) (33). Our transcriptome analysis identified several genes that were differentially expressed upon pD4-19 acquisition and whose expression remained altered upon adaptive evolution, arguing for intrinsic effects of the plasmid. Among those genes, several were associated with virulence and immune interference, including hemolysin genes (*hlgBC* and *hly*), the capsule biosynthesis operon *cap*, the serine protease locus *spl*, and the type VII secretion system *ess* (Fig. S10A). Also, transcription of the *icaADBC* operon was increased upon plasmid acquisition, and all genes except *icaD* remained highly expressed upon adaptive mutation. The *ica* operon allows the synthesis of the polysaccharide intercellular adhesin (PIA) and is thereby responsible for biofilm formation (Fig. S10A). In line with this, RN-T showed a significant increase in biofilm formation (Fig. 9). Interestingly, this phenotype was reverted upon adaptive evolution.

## DISCUSSION

Using our collection of nasal staphylococcal isolates, we identified the S. aureus strain D4-19 with a plasmid carrying the MP1 BGC. A number of studies have identified MP1 BGCs in a variety of staphylococcal species, including S. aureus (34), S. epidermidis (35), Staphylococcus equorum (36), Staphylococcus pseudintermedius (37), Staphylococcus agnetis, and S. hominis (20). Also, an isolate from Mammaliicoccus sciuri (formerly Staphylococcus sciuri) (38) has been shown to produce MP1. Moreover, the NCBI database includes various homologous sequences in S. aureus genomes as well as many coagulase-negative staphylococci. The abundance of this BGC in different strain backgrounds suggests intra- and interspecies horizontal transfer, which is supported by the fact that the MP1 gene clusters identified in staphylococci so far are all located on plasmids. The cellular consequences of BGC acquisition in staphylococci are profound, and we studied the reasons for metabolic constraints along with mechanisms overcoming them using evolutionary adaptation and multiomics approaches.

It is generally recognized that bacteriocin production is associated with metabolic costs for the producing cell and that expression is therefore frequently regulated, often in complex ways—for instance, depending on producer cell densities or on the detection of bacterial competitors (39, 40). Compound production generates metabolic burdens, as the responsible gene cluster needs to be propagated, precursors need to be channeled from the primary metabolism, and sufficient self-immunity needs to be established. This concept might be of special relevance for RiPPs such as MP1, as their production relies on cellular tRNA pools, and sudden expression upon acquisition of the BGC might therefore disturb central metabolic fluxes. It seems therefore likely that cellular fitness in the context of bacteriocin production requires adjustments of metabolic pathways. Our experiments showed that acquisition of the plasmid pD4-19 enabled production of bioactive MP1. However, the BGC recipient produced significantly less compound than the original plasmid host (S. aureus D4-19) while simultaneously displaying a growth defect compared to the plasmid-free parental strain RN4220.

Increased availability of citrate, of exogenous or endogenous origin, improved compound production and abrogated any growth defects. Interestingly, we found the recipient S. aureus RN4220 to carry a malfunctional *citZ* allele, limiting the levels of citrate synthase activity, of intracellular citrate and most likely, of the entire TCA cycle. Importantly, the entire lineage of S. aureus NCTC8325 (parental lineage of S. aureus RN4220) developed and maintained the malfunctioning *citZ* allele, and it has been observed previously that NCTC8325 enters stationary phase earlier than other S. aureus strains (41). Accordingly, it would be interesting to investigate if NCTC8325 (or RN4220 WT) would also benefit from an increased translation of CitZ.

However, the acquisition of pD4-19 made the metabolic shortcomings of RN4220 apparent. The most prominent phenotype of TCA cycle-deficient mutants is a growth defect that manifests after approximately 5 h of growth in glucose-containing medium (30). This is due to the fact that S. aureus preferentially degrades glucose via glycolysis or the pentose phosphate pathway and fermentation to produce acetate (29, 30). Concurrently, catabolite repression of glucose inhibits the TCA cycle (42). Only after glucose is consumed does acetate catabolism demand TCA cycle activity to create ATP and to sustain growth (30). S. aureus RN4220 does not show signs of TCA cycle deficiency. However, we observed that acquisition of pD4-19 and the associated MP1 production entailed premature growth arrest as well as increased levels of intracellular pyruvate and acetyl-CoA and extracellular accumulation of acetate, all of which are hallmarks of TCA cycle-deficient strains (30). Adaptive mutation reverted these signs of TCA deficiency. Acetyl-CoA levels dropped, and the levels of citrate and α-ketoglutarate increased suggesting efficient feeding of the TCA cycle. Simultaneously, accumulation of extracellular acetate was limited. Interestingly, we did not find that improvement of TCA cycle activity upon adaptive mutation increased the cellular ATP pools. In contrast, we found ATP levels in the adapted strain to be lower than in any other strain, despite the fact that the adapted strain showed a prolonged growth phase and produced more MP1 than the other strains. Accordingly, it is unlikely that increased needs for ATP upon plasmid acquisition were responsible for the observed growth deficiency of the original recipient strain RN-T.

It is tempting to speculate that MP1 production entailed excessive channeling of TCA cycle intermediates into the biosynthesis of amino acids, ultimately leading to premature growth arrest of the strain. Along this line, BGC acquisition had a wide impact on the metabolome of S. aureus, and adaptive mutation shifted the general pattern a second time to largely resemble that of the plasmid-free parental strain. This observation suggested that metabolic fluxes needed to be normalized by improvement of *citZ* expression and the associated increased availability of citrate. Interestingly, deletion of bacteriocin biosynthesis genes in enterococci has been associated with improved growth characteristics and altered expression of ribosomal proteins, vitamins, and glycolysis enzymes (43). Similarly, we found that restoration of the *citZ* allele also entailed a strong increase in expression of the translational machinery, cofactor biosynthesis, and the urea cycle, all suggesting an increase in protein biosynthesis and turnover, supporting the positive effect of the mutation on the cellular metabolism.

The impact of BGC acquisition on TCA cycle activity in S. aureus RN4220 is further underlined by the fact that the initial transformant produced high levels of biofilm, which was abrogated upon adaptive mutation in *citZ*. Several studies have associated TCA cycle activity with the staphylococcal capacity to form biofilms. A major factor determining staphylococcal biofilm formation is the production of the PIA (44, 45). We found the responsible *ica* genes to be strongly upregulated upon pD4-19 acquisition. Vuong et al. reported that reduced TCA cycle activity increased PIA production in S. epidermidis (46), and TCA-deficient mutants were shown to derepress the *ica* genes and to channel carbohydrates into PIA synthesis (47). We found strongly increased expression of *ica* genes upon pD14-19 acquisition, supporting the general finding that MP1 production induces a TCA-deficient phenotype in RN4220. Additionally, it was reported that citrate as well as TCA cycle intermediates can stimulate expression of the fibronectin-binding proteins FnbA and FnbB, resulting in *ica*-independent biofilm formation (48). However, we did not observe differences in *fnbA* and *fnbB* expression levels in our experiments, suggesting that this mechanism is not relevant for biofilm formation in S. aureus RN-T.

Restoration of the *citZ* allele also enhanced MP1 production by the RN4220 lineage. It is well accepted that expression of antibiotic BGCs in heterologous hosts can be limited due to an insufficient supply of appropriate cellular precursors. This is of special relevance for compounds produced by nonribosomal peptide synthetases or by polyketide synthases, which frequently rely on special precursors such as nonproteinogenic amino acids or unusual carbohydrates. For example, overexpression of rhamnose and forosamine biosynthetic pathways improved the biosynthesis of the polyketide antibiotic spinosad 1,000-fold (49), and medium optimization to provide appropriate precursors has proven to be an efficient strategy to enhance compound production (13). Similarly, optimization of the microbial central metabolic processes, including glucose, amino acid, and fatty acid metabolism, can boost compound production in heterologous hosts (50). For RiPPs like MP1, the relevance of this concept is less clear, as the compounds are produced using the ribosomal machinery, which relies on the cellular pool of canonical aminoacyl-tRNAs. This might ensure that precursor molecules are generally available and compound production is possible. However, it seems plausible that RiPP production can drain the pool of aminoacyl-tRNAs. This hypothesis is supported by the finding that addition of the amino acids glutamate, glycine, serine, and threonine as well as the addition of maltose enhanced the production of the RiPP gallidermin (51). However, our metabolomic analysis showed that, in the context of MP1 production, amino acid levels were not depleted in the BGC recipient at the time point of growth arrest, arguing against amino acid limitation as a sole underlying reason for the decreased compound production.

Finally, it has to be considered that effects that are independent of cellular metabolites might impact the fitness of antibiotic producers. For instance, production of the lantibiotics epidermin and gallidermin imposes a physiological burden on the producing staphylococcal strains because of insufficient immunity of the producer strains, leading to increased cell lysis (18). In contrast, we did not observe imperfect resistance of MP1-producing strains, suggesting that insufficient immunity does not substantially contribute to the observed growth defect of RN-T. The different findings for MP1 and epidermin are most likely due to the different modes of action and the associated resistance mechanisms. Lantibiotics target lipids I, II, and III in the bacterial membrane, ultimately damaging the integrity of the cell envelope and inducing lysis (18, 52). Resistance is imperfect and relies on the active expulsion of lantibiotics (53, 54). In contrast, MP1 targets the bacterial ribosome (21), and full resistance is provided by the expression of an alternative L11 ribosomal subunit (55). Interestingly, accumulation of amino acids similar to that observed in the pD4-19 recipient is known to be induced by antibiotic compounds targeting the bacterial ribosome (27). However, expression of the plasmid-associated resistance determinant did not change upon adaptive mutation in *citZ*, suggesting that altered levels of autoimmunity are not responsible for the observed increase of cellular fitness. However, it seems possible that the malfunctioning *citZ* allele might not be translated in the context of the alternative ribosomal subunit which might explain the TCA-deficient phenotype of the transformant. In general, our observations support a model in which pD4-19 acquisition and associated compound production drain the levels of TCA cycle intermediates and cause pleiotropic effects, including growth deficiency, reduced compound production, and increased biofilm formation. These findings enhance our understanding of how antibiotic production is integrated into and optimized by the cellular central metabolism. Interestingly, variation of TCA cycle activity is known to cause heterogeneity in staphylococcal populations (56), and TCA cycle-deficient mutants are reported to arise during invasive infection (57). These phenomena are known to cause formation of antibiotic-tolerant cells, and it also suggests that our findings might be relevant in staphylococcal strains in natural communities.

Even in environments such as the nasal microbiome, bacteriocin-producing strains remain rare, although the horizontal transfer of the responsible BGCs is possible, and competitive benefits of inhibiting competitors should be immense (1, 10). Our study can explain why most BGCs remain rare among isolates of a given bacterial species. As the consequence of a substantial metabolic burden imposed by bacteriocin production, a BGC-carrying mobile genetic element will most likely be counterselected against. Accordingly, BGCs will be maintained only if adaptive evolution events such as that reported in our study are possible and quickly occurring, while simultaneously, bacteriocin-sensitive competitors are efficiently cleared from the ecological niche. However, further experimental evidence is needed to support this idea.

## MATERIALS AND METHODS

### Strains and growth conditions.

The S. aureus strains used in this study were D4-19, RN4220 (RN), and USA300 LAC. S. aureus strains generated during this study were RN4220 pD4-19 (RN-T), RN4220 pD4-19 adapted (RN-A), D4-19 TN1/2/3, RN4220 pD4-19 ΔMP1, D4-19 ΔPP, and RN4220 pD4-19 ΔPP. Overexpression strains were constructed in the S. aureus RN4220 background carrying the plasmid pRB473-xylR-6×His-citZ, with *citZ* deriving from either S. aureus RN4220 pD4-19 or S. aureus RN4220 pD4-19 adapted. The construction of the plasmids and knockouts is described below. Escherichia coli DC10B or E. coli Sa08B was used as the cloning host for further transformation in S. aureus D4-19, RN4220, or RN4220 pD4-19.

BM (1% soy peptone A3 [Organotechnie SAS, France], 0.5% Ohly Kat yeast extract [Deutsche Hefewerke GmbH, Germany], 0.5% NaCl, 0.1% glucose, and 0.1% K_2_HPO_4_; pH 7.2) was used as the standard growth medium. If necessary, antibiotics were added at concentrations of 10 μg mL^−1^ for chloramphenicol, 2.5 μg mL^−1^ for erythromycin, and 0.5 μg mL^−1^ for penicillin G. E. coli transformants were grown in lysogeny broth (LB; Lennox) medium (1% tryptone, 0.5% yeast extract, and 0.5% NaCl; Carl Roth GmbH, Germany) supplemented with 10 μg mL^−1^ chloramphenicol or on BM agar with 10 μg mL^−1^ chloramphenicol. BM without glucose (B_0_) was used for expression of *citZ*.

To monitor growth over time, strains were grown overnight in BM with continuous shaking at 37°C. Each strain was adjusted to an OD_600_ of 1 in BM, and 5 μL of the bacterial stock solutions was pipetted into 1 mL BM into a 24-well microtiter plate. If necessary, 5 mM sodium citrate was added to each well. The plates were incubated for 24 h with continuous shaking in a microplate reader, and OD_600_ was measured every 15 min.

For all experiments assessing growth or transcription or metabolic profiles, bacterial strains were grown in 10-mL volumes of medium in 50-mL Erlenmeyer flasks containing a single baffle. Cultures were incubated at 37°C with 160 rpm agitation. CO_2_ levels were not controlled.

### Cloning.

DNA manipulation, isolation of plasmid DNA, and transformation of E. coli and S. aureus were performed by using standard procedures. Enzymes for molecular cloning were obtained from Thermo Fisher Scientific.

### Transposon mutagenesis.

To identify the biosynthetic gene cluster responsible for antimicrobial activity of S. aureus D4-19, the strain was transformed with the plasmid pBTn and mutants were generated by transposon insertion as described previously (58). To identify the insertion site of the transposon in clones that had lost antimicrobial activity, genomic DNA was isolated, and an inverse PCR was performed. For this, 5 μg genomic DNA (gDNA) was digested with the restriction enzyme BspHI for 3h at 37°C. After purification of the digest, 2 μg of DNA was religated in 100 μL total volume for 2 to 3 h and 2 μL of the ligation mixture was used for standard PCR (in a 25-μL volume) with the primers pBTn up and pBTn down (Table 1). The PCR products were analyzed on an analytical gel; strong bands were isolated and sequenced with the primers pBTn up and pBTn down.

**TABLE 1:** Primers used for transposon mutagenesis and generation of knockout mutants

Primer	Sequence (5′–3′)	Use
pBTn seq up	ACCAACATGACGAATCCCTCC	Inverse PCR and sequencing
pBTn seq down	CCCGCCATACCACAGATGTT	Inverse PCR and sequencing
TclS	GCATCAGACACCTGTTCACTTATT	pD4-19 sequencing
BlaR1-fwd	CTATGGCTGAATGGGATGTTAT	pD4-19 sequencing
WT_KO_PP_up_fwd	CGAGGGTCGACTAAACTTAAACCTGACTGTCATTGT	Deletion of MP1 precursor peptide (*tclE*) and MP1 BGC
WT_KO_PP_up_rev	CCTAACTAAATTATTATTACGAGCACCACCTTTACTTAGAT	Deletion of MP1 precursor peptide (*tclE*)
WT_KO_PP_dw_fwd	GGTGGTGCTCGTAATAATAATTTAGTTAGGTATAAATTA	Deletion of MP1 precursor peptide (*tclE*)
WT_KO_PP_dw_rev	CTGCAGGAATTCAGAATATCTAGTATCGAAGATTT	Deletion of MP1 precursor peptide (*tclE*)
KO_MP1_up_rev	TTTAATATAATTGTATGGAGGTTAGTTACGAGCACCACCTTTACTTAGAT	Deletion of MP1 BGC
KO_MP1_down_fwd	ATCTAAGTAAAGGTGGTGCTCGTAACTAACCTCCATACAATTATATTAAA	Deletion of MP1 BGC
KO_MP1_down_rev	ATATGAATTCATCGAGTTGTCGAAATGTTAGAA	Deletion of MP1 BGC
pTX15 Hind	CGTTATCACAAGTGGTCACCACTCT	Construction of pRB473-xylR
pTX15 Sma	GCTTCCGGCTCGTATGTTGTGTGG	Construction of pRB473-xylR
His-term down	GGCTAGCGGATCCTCGAGTCGACTACCGAGCTCAGATCTCATC	Insertion if 6xHis in pRB473-xylR
His-term up	AGTTAGAATTCTGCAGTTTCATGAATATTACAAACAAAAAGC	Insertion if 6xHis in pRB473-xylR
citZ_overex_fwd	AATGTAGGATCCAATTGTTGCACATATAATGCCAGTT	Amplification of *citZ* with promoter region
citZ_overex_rev_new	AGATCTGAGCTCTTTTCTTTCTTCAAGCGGGATATAC	Amplification of *citZ* with promoter region

### Generation of S. aureus RN4220 pD4-19 and adapted mutant.

S. aureus D4-19 belongs to clonal complex 8, and, to circumvent restriction-modification (RM) barriers, we used the laboratory strain S. aureus RN4220 as the recipient strain. This RM-deficient strain belongs to the same clonal complex, exhibits high transformation rates, and does not show inhibitory effects on other staphylococci. Finally, S. aureus RN4220 is highly sensitive to penicillin G (MIC = 0.015 μg/mL), allowing the selection of pD4-19, which encodes a β-lactamase. Plasmid DNA was isolated from S. aureus D4-19 and was used to transform S. aureus RN4220 by electroporation. Positive transformants were selected on BM plates containing 0.5 μg mL^−1^ penicillin G and screened by PCR with primers binding in the BGC and the β-lactamase gene cluster (Table 1). Plasmid isolation and restriction digestion confirmed that S. aureus RN4220 had acquired the plasmid pD4-19 and propagated it as an extrachromosomal element.

To generate a mutant that had adapted to MP1 production, S. aureus RN4220 pD4-19 was passaged for 28 days by inoculating fresh BM every day with 10 μL of overnight culture.

### Generation of knockout mutants.

For the generation of knockout mutants, the temperature-sensitive shuttle vector pIMAY was used, and mutants were generated by allelic replacement as described previously (59). Flanking regions of the genes to be deleted were amplified by PCR (Table 1) and ligated into pIMAY after digestion with suitable restriction enzymes. Cloning was performed in E. coli DC10B or E. coli Sa08B. Sequence-verified plasmids were transferred in the target strains, S. aureus D4-19 and S. aureus RN4220 pD4-19. Successful knockouts were confirmed by PCR with respective primers and sequencing of the PCR product covering the area of the knocked-out gene.

### Overexpression of *citZ*.

To overexpress *citZ*, a novel xylose-inducible plasmid was constructed on the basis of the shuttle vector pRB473. The regulatory unit containing the *xylR* repressor gene and about 200 nucleotides at its 3′ end encompassing the regulated promoter were amplified with primers pTX15 Hind and pTX15 Sma and plasmid pTX15 as the template (Table 1). From the resulting PCR product, the required fragment was generated via HindIII and BamHI digestion, which was subsequently ligated to pRB473 digested in a similar manner, resulting in pRB473-xylR. Furthermore, a 6×His tag was inserted into the plasmid to obtain a C-terminally tagged protein. Therefore, plasmid pPSHG3 was used as the template to amplify the 6×His tag with the primers His-term down and His-term up (Table 1). The insert and pRB473-xylR were digested with EcoRI and BamHI, and after ligation, E. coli DC10B was transformed. *citZ* was amplified from S. aureus RN4220 pD4-19 and S. aureus RN4220 pD4-19 adapted using the primers citZ_overex_fwd and citZ_overex_rev_new (Table 1). After restriction digestion with BamHI and SacI, the inserts were ligated into pRB473-xylR-6×His. Sequence-verified plasmids were transferred into S. aureus RN4220.

To check the expressed protein levels, strains were grown overnight in BM, inoculated to an OD_600_ of 0.1 in B_0_, and grown to an OD_600_ of 0.5. After induction with 0.5% xylose, samples were taken at 0 h, 2 h, and 4 h. Samples were loaded on an SDS-PAGE gel (12% precast gel; Bio-Rad) after incubation with 4× Laemmli sample buffer (Bio-Rad; 355 mM 2-mercaptoethanol added) at 95°C for 5 min and centrifugation for 10 min at 16 000 × *g*. A PageRuler prestained protein ladder (Thermo Scientific) was used, and the gel was run at 120 V for 1 h. For Western blotting, a nitrocellulose membrane was cut to the size of the gel and the blotting paper, and the membranes were equilibrated in transfer buffer. Blotting paper, gel, membrane, and blotting paper were stacked in a blotting chamber of the Bio-Rad Transblot Turbo system.

To detect the His tag, the membrane was incubated in blocking buffer and then washed with phosphate-buffered saline–Tween (PBS-T) and PBS. After incubation with the primary antibody from Qiagen (Penta-His antibody, 1:1,000 dilution) at room temperature (RT) for 1 h with shaking, the membrane was washed again, three times with PBS-T and once with PBS. The secondary antibody from LI-COR (IRDye 680RD goat anti-rabbit IgG, 1:10,000 dilution) was added and, after incubation for 1 h at RT with shaking, washed again as described above. The membrane was analyzed using a LI-COR Odyssey system. Protein amounts were calculated using the LI-COR Odyssey imaging tools.

### MIC assay.

Strains used for MIC determinations were grown overnight in BM with continuous shaking at 37°C. Each strain was adjusted to an OD_600_ of 0.00125 in BM, and 200 μL was pipetted into the first well of each row of a 96-well microtiter plate. One hundred microliters was pipetted into the remaining wells; one well per row contained 100 μL BM as a blank control. The MP1 stock solution was serially diluted in this 96-well microtiter plate, and the plates were incubated at 37°C for 21 h with continuous shaking (160 rpm). The OD_600_ of cultures in each well was measured with a microplate reader, and the lowest concentration of MP1 leading to no bacterial growth was defined as the MIC.

### Citrate assay.

Citrate levels were measured using the citrate assay kit (MAK057) from Sigma-Aldrich. Strains were grown overnight in BM, diluted into fresh BM to an OD of 0.1, and grown for 5 h at 37°C with continuous shaking. For each strain, 1 × 10^8^ cells in 100 μL citrate assay buffer were homogenized in a 1.5-mL microcentrifuge tube containing 100 μL glass beads with a FastPrep instrument at 6.5 m/s for 60 s. After centrifugation for 10 min at maximum speed, 30 μL of supernatant was pipetted into a 96-well plate. Twenty microliters of citrate assay buffer was added to reach the final volume of 50 μL described in the kit manual. Reaction mixes were prepared, and the analysis was carried out as described in the manufacturer’s manual.

### Acetate assay.

Acetate levels were measured using the acetate colorimetric assay kit (MAK086) from Sigma-Aldrich. Strains were grown overnight in BM, fresh BM was inoculated to an OD of 0.1, and strains were grown for 8 h at 37°C with continuous shaking. At 0, 2, 4, 6, and 8 h, 1 mL of culture was centrifuged at 11,000 × *g* for 5 min, and the supernatant was transferred into a fresh tube. Samples were diluted 1,000-fold, reaction mixes were prepared, and the analysis was carried out as described in the manufacturer’s manual.

### DNA isolation and sequencing.

DNA isolation, library preparation and sequencing were performed by the Institute for Medical Microbiology (part of the NGS Competence Center NCCT, Tübingen, Germany).

DNA was extracted using the Qiagen 20/G genomic tip kit, following the manufacturer’s instructions. The genomic DNA was quantified with a Qubit double-strand DNA (dsDNA) broad-range (BR) assay kit (Thermo Fisher).

Oxford Nanopore Technologies (ONT) library preparation was performed following the instruction manual. Native barcoding genomic DNA (with EXP-NBD196 and SQK-LSK109; Oxford Nanopore) was used, with an input of 250 ng DNA. Twelve microliters of template DNA was supplemented with the required reagents from the NEBNext Ultra II end repair/dA tailing kit (E7546S; New England Biolabs [NEB]) and was incubated first at 20°C for 5 min and then at 65°C for 5 min. For the barcode ligation, 3 μL of nuclease-free water, 0.75 μL end-prepped DNA, 1 μL native barcode (native barcoding expansion 96; EXP-NBD196), and 5 μL blunt/TA ligase master mix (M0367; NEB) were combined in a new reaction vessel and incubated for 20 min at room temperature. One microliter of 0.5 M EDTA was added, and samples were pooled in a new reaction tube. The pool was cleaned up by using AMPure XP beads (Agencourt), washed twice with 70% ethanol, and resuspended in nuclease-free water. For barcode ligation 5 μL adapter mix II, 10 μL NEBNext quick ligation reaction buffer (5×), and 5 μL quick T4 DNA ligase were added to the pool and incubated for 10 min at room temperature. The pool was cleaned up using AMPure XP beads, washed twice with long fragment buffer, and eluted in elution buffer. The library pool was loaded on a MinION device (ONT) and stopped at 39 Gb output. Base calling was performed using ONT’s Guppy base caller version 4.1.1.

Libraries for Illumina short-read sequencing were prepared using the “Illumina Nextera DNA Flex library preparation kit” (Illumina) with “IDT for Illumina DNA/RNA UD indexes, tagmentation” (Illumina) according to the manufacturer’s protocol with 500 ng DNA input, and 5 cycles of indexing PCR. Libraries were checked for correct fragment length on an Agilent 2100 Bioanalyzer, pooled equimolarly, and quantified with a Qubit DNA high sensitivity (HS) assay kit (Thermo Fisher). Equimolarly pooled libraries were sequenced on a MiSeq reagent kit v2 (300 cycles) flow cell (Illumina) with 2 × 150 bp read length. For demultiplexing, bcl2fastq v2.19.0.316 was used (https://emea.support.illumina.com/downloads/bcl2fastq-conversion-software-v2-20.html).

### DNA data assessment and analysis.

Sequencing statistics, including the quality per base and adapter content assessment of Illumina reads, were conducted with FastQC v0.11.8 (http://www.bioinformatics.babraham.ac.uk/projects/fastqc; accessed June 2022). Unicycler v0.5.0 (60) with default parameters was used for a hybrid assembly of the Oxford Nanopore and Illumina reads of the S. aureus RN4220 pD4-19 genome. The resulting genome was annotated using Prokka v1.14.6 (61) with the additional parameters to add gene features in the annotation and search for noncoding RNAs (parameters –addgenes and –rfam). The quality of the assembly was assessed using QUAST v5.1.0 (62). The genome of the Illumina reads of S. aureus RN4220 pD4-19 adapted was assembled using EAGER v1.92.56 (63) and MUSIAL v1.0 (https://github.com/Integrative-Transcriptomics/MUSIAL/tree/v1.0). As a reference the assembly of S. aureus RN4220 pD4-19 was used. In EAGER, parameters were set to not merge the paired-end reads and to use bwa-mem (64) for the mapping. For SNP calling, GATK HaplotypeCaller was chosen (65).

Whole-genome sequence of S. aureus D4-19 was determined by Illumina short-read sequencing as described above. Illumina reads were *de novo* assembled with SPAdes (version: 3.9.0) (66), and the plasmid contig was identified with MAUVE (67). The MP1 BGC was analyzed with antiSMASH 5.0 (bacterial settings) (68).

### Spot assay.

Antimicrobial activity was assessed, by resuspending the sensitive S. aureus USA300 LAC in 200 μL BM and spreading it with a cotton swab on a BM plate. Producer strains were also resuspended in BM and 10 μL of the suspension were spotted on the prepared indicator plate. Once the spots were dry, the plates were incubated at 37°C overnight.

### Purification of MP1.

Cell-bound MP1 was isolated as follows. Fifty milliliters of overnight culture was centrifuged at 6,000 × *g* for 10 min, and the pellet was washed twice with 15 mL PBS and then resuspended in 3 mL methanol. After incubation on a spinning wheel for 1 h and a centrifugation step at 6,000 × *g* for 10 min, the supernatant was transferred to a fresh Falcon tube and used for HPLC or MS/MS analysis.

### LC-MS analysis of MP1.

LC-MS analyses were performed with an HPLC (Ultimate 3000; Thermo Fisher) and subsequent HR-ESI(+)-TOF-MS (Maxis 4G; Bruker). For HPLC analysis, LC-MS-grade water (with 0.01% formic acid) and LC-MS-grade methanol (with 0.06% formic acid) were used, and fractionation was performed with a gradient from 10% to 100% over 20 min and a flow rate of 0.3 mL/min. A Nucleoshell RP 18 column with a length of 150 mm, an inner diameter of 2 mm, and a particle size of 2.7 μm, prewarmed to 40°C, was used. For HR-ESI(+)-TOF-MS, sodium formate was used as the calibrant.

### NMR analysis of MP1.

NMR analyses were performed on a Bruker AvanceIII-700 instrument. ^1^H NMR spectra were recorded with a frequency of 700 MHz, and ^13^C NMR spectra were recorded with a frequency of 176 MHz, both at a temperature of 303 K.

### Sample preparation for metabolome analysis.

For the preparation of samples for metabolome analysis, strains were grown over night, inoculated in fresh medium (20 mL) to an OD_600_ of 0.1, and grown for 5 h at 37°C with continuous shaking. OD_600_ was measured, and all strains were set to the lowest OD measured. The culture was filtered through a 0.22-μm bottle-top sterile filter (250 mL; Nalgene) via vacuum. The filter was washed with 0.6% ice-cold NaCl, cut into 4 pieces, and incubated at −20°C for 20 min with 5 mL ice-cold 40:40:20 (vol/vol/vol) methanol-acetonitrile-water in a glass bottle. One milliliter of filtrate was transferred to a microcentrifuge tube and bead beaten twice for 30 s each time at 6.5 m/s with 0.5 mL glass beads. Samples were kept on ice between runs. After centrifugation for 5 min at 4°C at maximum speed, 600 μL of supernatant was stored at −80°C.

### Metabolite analysis by FI-MS.

Metabolites were analyzed by flow injection into a high-resolution quadrupole time-of-flight (QTOF) mass spectrometer (Agilent QTOF 6546) as described previously (69). Three microliters of the sample was injected with an Agilent 1290 Bio Multisampler (G7137A) into the mobile phase, which was a 60:40 (vol/vol [percent]) mixture of isopropanol (LiChrosolv Supelco hypergrade for LC-MS; 1.02781.2500) and ultrapure water (Omnia Pure; Stakpure), buffered with 10 mM ammonium carbonate [(NH_4_)_2_CO_3_] (Sigma-Aldrich, 3799-10 G) and 0.04% ammonium hydroxide (NH_4_OH) (Honeywell/Fluka TraceSELECT Ultra, 16748-250 ML). The flow rate of the mobile phase was 0.15 mL min^−1^. Mass spectra were separately recorded in positive and negative ionization profile modes from *m/z* 50 to *m/z* 1,700 with an acquisition rate of 1.4 ms/spectrum using the highest resolving power (10 GHz; high sensitivity). Online mass axis correction was performed with purine and hexakis(1H,1H,3H-tetrafluoropropoxy)phosphazene (HP-0921; Agilent Technologies). The source gas temperature of the ESI ion source was 225°C, with 11 L min^−1^ drying gas and a nebulizer pressure of 20 lb/in^2^. The sheath gas temperature was 350°C, and the flow rate was 10 L min^−1^. Electrospray nozzle and capillary voltages were 2,000 and 3,500 V, respectively. Fragmenter and skimmer voltages were 120 and 65 V, respectively. Ion peaks were annotated by matching the mass-to-charge ratios to calculated, single (de)protonated masses of metabolites listed in a genome-scale model of Escherichia coli K-12 (iML1515) (70). A hierarchical cluster plot was generated using MATLAB. Metabolites obtained via FI-MS, which were used for further analysis, are listed in Table 2.

**TABLE 2:** Metabolites and their isobars measured via FI-MS

Metabolite(s)	*m*/*z*
Citrate/isocitrate/5-dehydro-4-deoxy-d-glucarate [M−H]^−^	191.020
α-Ketoglutarate [M−H]^−^	145.014
Malate [M−H]^−^	133.014
Fumarate [M−H]^−^	115.004
Pyruvate [M−H]^−^	87.009
Lactate/glyceraldehyde/dihydroxyacetone [M−H]^−^	89.024
Acetate [M−H]^−^	59.014
Glucose, galactose, allose, fructose, mannose and *myo*-inositol [M−H]^−^	179.056
Citrulline [M+H]^+^	176.103
Butyrate [M+H]^+^	89.060
Carnitine [M+H]^+^	162.112
Choline [M+H]^+^	104.107

### Metabolite analysis by LC-MS/MS.

LC-MS/MS was performed with an Agilent 6495 triple-quadrupole mass spectrometer (Agilent Technologies) as described previously (71). An Agilent 1290 Infinity II ultra-high-performance liquid chromatography (UHPLC) system (Agilent Technologies) was used for liquid chromatography using two columns: (i) an Acquity ultraperformance liquid chromatography (UPLC) ethylene-bridged hybrid (BEH) amide (Waters) for acidic conditions and (ii) an iHILIC-Fusion(P) (HILICON AB) for basic conditions. The column oven was at 30°C. LC solvents were as follows: solvent A was water with ammonium formate (10 mM) and formic acid (0.1% [vol/vol]) for acidic conditions and water with ammonium carbonate (10 mM) and ammonium hydroxide (0.2%) for basic conditions; solvents B was acetonitrile with formic acid (0.1% [vol/vol]) for acidic conditions and acetonitrile for basic conditions. The LC gradient was as follows: 0 min, 90% B; 1.3 min, 40% B; 1.5 min, 40% B; 1.7 min, 90% B; 2 min, 90% B. The flow rate was 0.4 mL/min. The injection volume was 3 μL. Settings of the ESI source were as follows: 200°C source gas, 14 L/min drying gas, and 24 lb/in^2^ nebulizer pressure. The sheath gas temperature was at 300°C, and the flow rate was 11 L/min. The electrospray nozzle was set to 500 V and capillary voltage to 2,500 V. All samples were mixed with a ^13^C-labeled internal standard and the ratio of ^12^C and ^13^C peak heights was used to quantify metabolites. ^12^C/^13^C ratios were normalized to the OD-specific cell volume at the time point of sampling. Alanine, arginine, asparagine, aspartate, glutamate, glutamine, glycine, histidine, leucine/isoleucine, lysine, methionine, phenylalanine, proline, serine, threonine, tyrosine, tryptophan, valine, acetyl-CoA, succinate, 3-phosphoglycerate, phosphoenolpyruvate, urea, ornithine, argininosuccinate, and ATP were measured via LC-MS/MS.

### Sample preparation for proteome analysis.

Strains were grown overnight, inoculated in fresh medium to an OD_600_ of 0.1, and grown for 5 h at 37°C with continuous shaking. A 1.5-mL portion was centrifuged, and the pellet was resuspended in 1 mL SDS buffer (4% [wt/vol] sodium dodecyl sulfate [SDS] in 100 mM Tris-HCl [pH 8]). Cells were homogenized at 6.5 m/s twice for 40 s each time by using a FastPrep and incubated on ice for 2 min between steps. Samples were centrifuged at maximum speed for 1 min, and the supernatant was transferred into a fresh Eppendorf tube. To reduce cysteine disulfide bonds, 10 mM dithiothreitol (DTT) was added to the samples and incubated for 45 min with shaking at 650 rpm at RT. Iodoacetamide (IAA; 5.5 mM) was added to alkylate reduced cysteine thiol groups. Incubation for 45 min at RT with shaking at 650 rpm in the dark was followed by centrifugation of the samples at 12 000 × *g* for 15 min. The supernatant was transferred in a new tube, and 1 volume of supernatant was mixed with 7 volumes of ice cold 8:1 acetone-methanol, vortexed, and incubated overnight at −20°C. Centrifugation of the precipitated proteins for 5 min at 1,000 × *g* was followed by two washing steps with 80% acetone at RT. The protein pellet was air dried for 10 to 15 min and rehydrated in denaturation buffer (6 M urea, 2 M thiourea in 10 mM Tris-HCl; pH 7.5).

### LC-MS/MS analysis of proteome samples.

Ten micrograms of proteins per sample was digested in solution with trypsin as described previously (72). Desalted peptides (73) were separated on an Easy-nLC 1200 system coupled to a quadrupole Orbitrap Exploris 480 mass spectrometer (all Thermo Fisher Scientific) as described previously (74) with slight modifications: peptides were separated using an 87-min segmented gradient of 10, 33, 50, and 90% HPLC solvent B (80% acetonitrile in 0.1% formic acid) in HPLC solvent A (0.1% formic acid) at a flow rate of 200 nL/min. The mass spectrometer was operated in data-dependent mode, collecting MS spectra in the Orbitrap mass analyzer (60,000 resolution, 300-to-1,750 *m/z* range) with the automatic gain control (AGC) set to standard and the maximum ion injection time set to automatic. The 20 most intense precursor ions were sequentially fragmented with a normalized collision energy of 28 in each scan cycle using higher-energy collisional dissociation (HCD) fragmentation. In all measurements, sequenced precursor masses were excluded from further selection for 30 s. MS/MS spectra were recorded with a resolution of 15,000, with fill time set to automatic. Acquired MS spectra were processed with the MaxQuant software package version 1.6.14.0 (75) with an integrated Andromeda search engine (76).

A database search was performed against a Staphylococcus aureus (allStrains) protein database (downloaded on 7 October 2020; 216,059 entries) and 286 commonly observed contaminants. Endoprotease trypsin was defined as protease with a maximum of two missed cleavages. Oxidation of methionine, and protein N-terminal acetylation were specified as variable modifications. Carbamidomethylation on cysteine was set as a fixed modification. The initial maximum allowed mass tolerance was set to 4.5 ppm for precursor ions and 20 ppm for fragment ions. Peptide, protein, and modification site identifications were reported at a false discovery rate (FDR) of 0.01, estimated by the target-decoy approach (77). The iBAQ (intensity-based absolute quantification) and LFQ (label-free quantification) algorithms were enabled, as was the “match between runs” option (78, 79).

### RNA isolation for transcriptome analysis.

Strains were grown overnight, inoculated in fresh medium to an OD_600_ of 0.1 and grown for 5 h at 37°C with continuous shaking. A 1/10 volume of ethanol (EtOH)-phenol was added to 500 μL of sample and mixed for 1 min. After incubation on ice for 5 min, the samples were centrifuged for 1 min at 20,000 × *g* at 4°C. The supernatant was discarded, and the pellet was resuspended in 1 mL TRIzol. Each sample was transferred to one screw-cap tube with glass beads, and cells were lysed via bead-beating twice at 6.5 m/s for 30 s each time. Between the two runs, the cells were kept on ice for 2 min. Two hundred microliters of chloroform was added to the samples, and samples were mixed and incubated 2 to 3 min before centrifugation for 15 min at 12,000 × *g* and 4°C. The aqueous supernatant was mixed with 500 μL isopropanol, and samples were centrifuged for 10 min at 21,000 × *g* and 4°C. The supernatant was discarded, and the pellet was resuspended in 500 μL 75% EtOH. After centrifugation for 5 min at 20,000 × *g* and 4°C, the supernatant was discarded, and the pellet was dried at RT. The pellet was resuspended in 100 μL RNA-grade water, and RNA was concentrated with the MN RNA clean up kit (Macherey-Nagel). RNA was eluted in 60 μL RNA-grade water. After quantification with a NanoDrop spectrophotometer, RNA was stored at −80°C. Library preparation and sequencing were performed by the Institute for Medical Microbiology (part of the NGS Competence Center NCCT [Tübingen, Germany]). RNA samples were DNase I digested (DNase I recombinant, RNase free; Millipore Sigma), cleaned up (RNA Clean & Concentrator-5; Zymo Research), quantified (Qubit RNA BR assay kit; Thermo Fisher), and normalized to 100 ng in 11 μL nuclease-free water. Library preparation was performed according to the Illumina reference guide for stranded total RNA preparation and ligation with Ribo-Zero Plus. Library concentration was measured with a Qubit DNA HS assay kit (Thermo Fisher) on a Qubit fluorometer (Invitrogen), and fragment length was assessed with an Agilent 2100 Bioanalyzer (Agilent high-sensitivity DNA kit). Samples were equimolarly pooled and sequenced with a NextSeq 500 high-output kit v2.5 (75 cycles) flow cell (Illumina) with 1 × 75 bp read length.

### RNA-Seq data assessment and analysis.

Sequencing statistics, including the quality per base and adapter content assessment of resulting transcriptome sequencing data, were obtained with FastQC v0.11.8 (http://www.bioinformatics.babraham.ac.uk/projects/fastqc; accessed June 2022). All read mappings were performed against the previously assembled reference strain S. aureus RN4220 pD4-19 (SRA BioProject ID PRJNA855446). The mappings of all samples were conducted with HISAT2 v2.1.0 (80). As parameters, spliced alignment of reads was disabled and strand-specific information was set to the reverse complemented (HISAT2 parameter –no-spliced-alignment and –rna-strandness “R”). The resulting mapping files in SAM format were converted to BAM format using SAMtools v1.9 (81). Mapping statistics, including strand specificity estimation and percentage of mapped reads, were conducted with the RNA-Seq module of QualiMap2 v2.2.2-a (82). Gene counts for all samples were computed with featureCounts v1.6.4 (83) based on the Prokka annotation of the assembled S. aureus RN4220 pD4-19 genome, where the selected feature type was set to transcript records (featureCounts parameter -t transcript). A quality check for rRNA was performed with an in-house-written script based on the absolute counts of annotated rRNAs. To assess variability of the replicates of each time series, a principal-component analysis (PCA) was conducted with the DESeq2 package v1.28.1 (84).

### Normalization and differential gene expression.

For the computation of genes differentially expressed between the two different strains (S. aureus RN4220 pD4-19 and pD4-19 adapted) and the wild-type strain S. aureus RN4220, DESeq2 v1.20.0 (84) was applied to the absolute gene counts as computed with featureCounts. For differences between the two strains and the wild-type strain, genes with an adjusted *P* value (FDR) of <0.05 and absolute log_2_ fold change (FC) of >1 were reported as differentially expressed.

### Gene set enrichment analysis.

The assembled S. aureus RN4220 pD4-19 genome was functionally annotated using FACoP (http://facop.molgenrug.nl/). Gene set enrichment analysis was performed on differentially expressed genes using FUNAGE-Pro (http://gseapro.molgenrug.nl/).

### Biofilm assay.

Strains were grown over night in tryptic soy broth and adjusted to the OD_600_ of the strain with the lowest OD_600_. Five microliters was added to 995 μL of BM containing 1% ([vol/vol]) glucose. Two hundred microliters was transferred to the wells of a fibrinogen-coated Nunclon Delta surface microtiter plate. The plate was incubated at 37°C for 24 h without shaking. Control wells with broth and no bacteria were included. The supernatant was discarded, and wells were washed three times with 200 μL PBS. The plate was inverted for 30 min to dry. One hundred microliters of crystal violet was added to each well and washed off after 1 min (3 to 5 times with PBS). One hundred microliters of 5% acetic acid was added to the wells, and the plate was placed on a shaker for 5 min to dissolve the cells. Subsequently the absorbance at 570 nm was measured using the FLUOstar Optima reader.

### Statistical analysis.

Statistical analyses were performed using GraphPad Prism 9.02.

### Data availability.

ONT and Illumina reads for S. aureus RN4220 pD4-19 and S. aureus RN4220 pD4-19 adapted can be found at SRA BioProject ID PRJNA855446 and the plasmid sequence of pD4-19 (from S. aureus D4-19) has the GenBank accession number ON936820. All RNA-Seq Illumina read files as well as the raw counts have been deposited in NCBI’s Gene Expression Omnibus and are accessible under accession number GSE208001. Metabolome data can be accessed at MetaboLights via the identifier MTBLS5196. Proteome data can be accessed at PRIDE via the accession number PXD035193.
