# Supplementary material for: Horizontal Transfer of Bacteriocin Biosynthesis Genes Requires Metabolic Adaptation To Improve Compound Production and Cellular Fitness
Source: Microbiol Spectr. 2022 Dec 6;11(1):e03176-22. doi: 10.1128/spectrum.03176-22 (PMC9927498; doi:10.1128/spectrum.03176-22)
Supplement: Supplemental file 1 — Supplemental material. Download spectrum.03176-22-s0001.pdf, PDF file, 0.9 MB [file spectrum.03176-22-s0001.pdf]

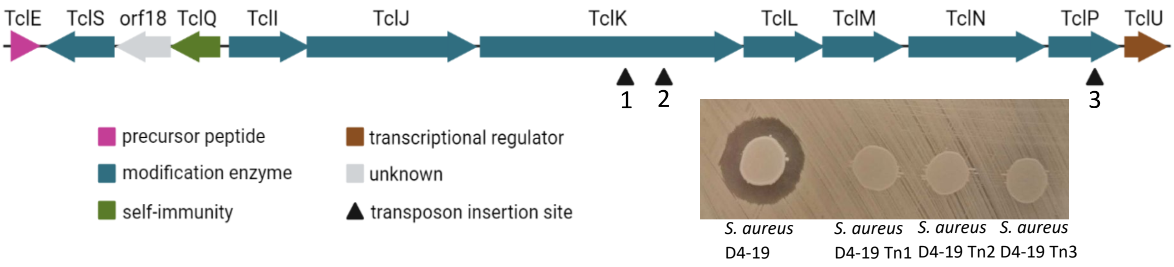

Figure S1: Transposon insertion sites in the MP1 gene cluster of *S. aureus* D4-19 as indicated by arrowheads and spot assay of these mutants on *S. aureus* USA300 LAC to confirm loss of antimicrobial activity.

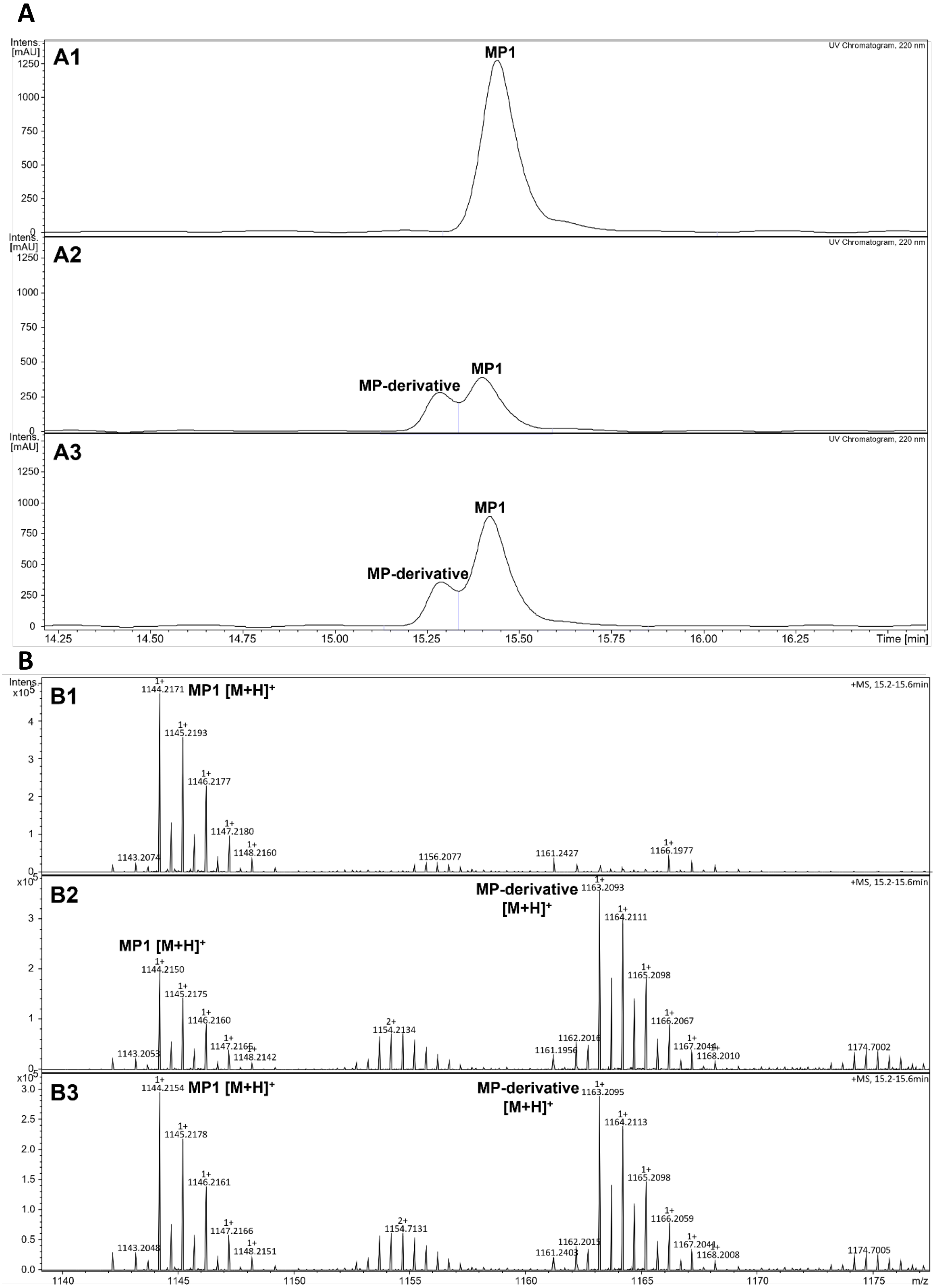

Figure S2: A) HPLC-UV-chromatograms of extracts of *S. aureus* D4-19 (A1), *S. aureus* RN-T (A2) and RN-A (A3). **B)** Mass spectra of micrococcins (MP1 ($[M+H]^+$, calculated m/z = 1144.2173) and MP-derivative ($[M+H]^+$, calculated m/z = 1163.2116)) detected in *S. aureus* D4-19 (B1), *S. aureus* RN-T (B2) and RN-A (B3).

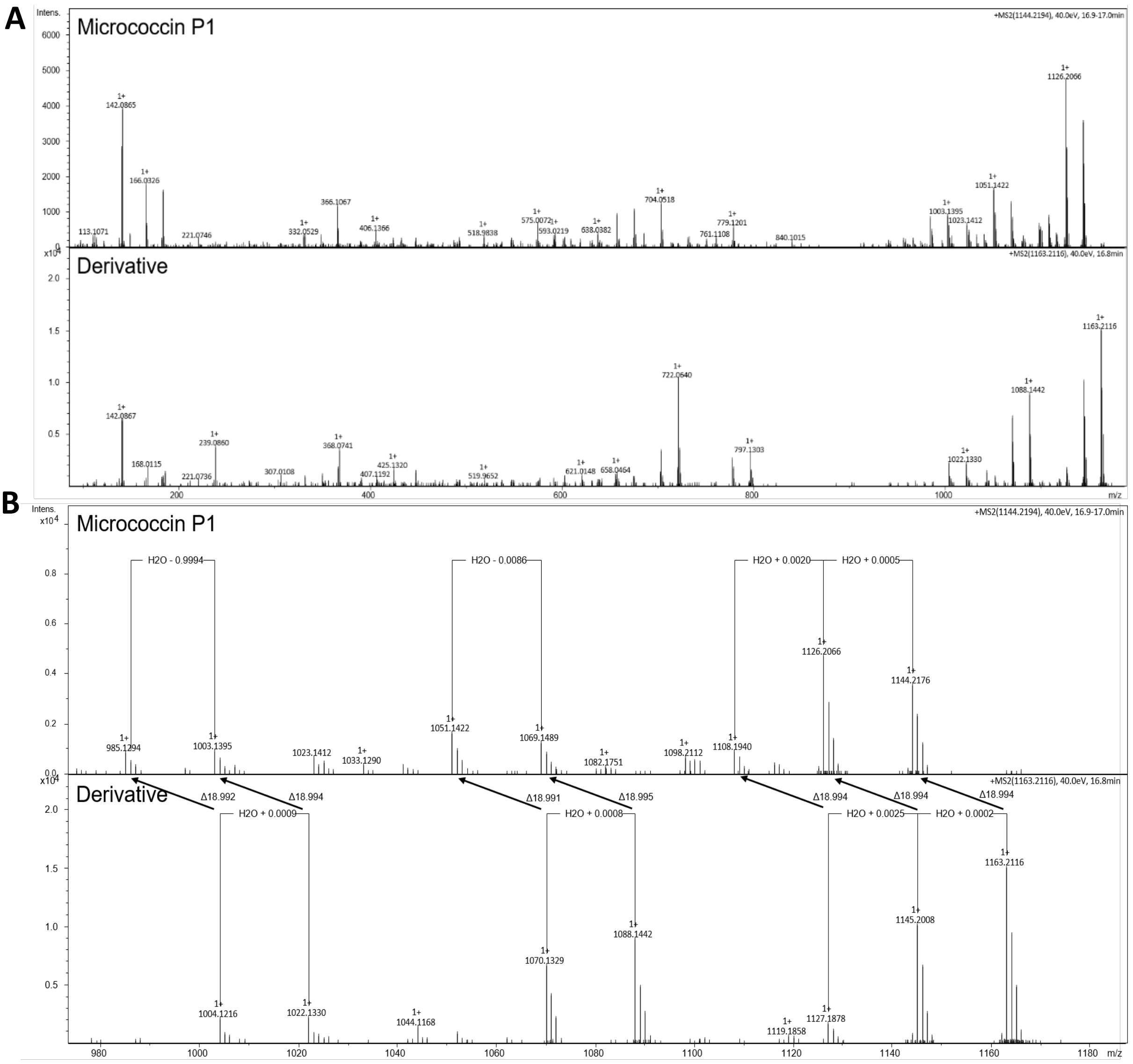

Figure S3: High performance liquid chromatography coupled tandem mass spectra (HPLC-ESI(+)-MS/MS) of MP1 and MP-derivative. **A)** Full tandem mass spectra (MS/MS) of MP1 and the derivative. **B)** Tandem mass spectra (MS/MS) of MP1 ([M+H]$^+$, m/z = 1144.2171 (found), 1144.2173 (calculated for $C_{48}H_{50}N_{13}O_9S_6$) and MP-derivative (m/z = 1163.2116) with focus on the first fragmentations showing losses of water and mass differences between both compounds. Tandem mass spectra were recorded with high performance liquid chromatography coupled high resolution electrospray mass spectrometer with time-of-flight detection (HPLC-HR-ESI(+)-TOF-MS).

A

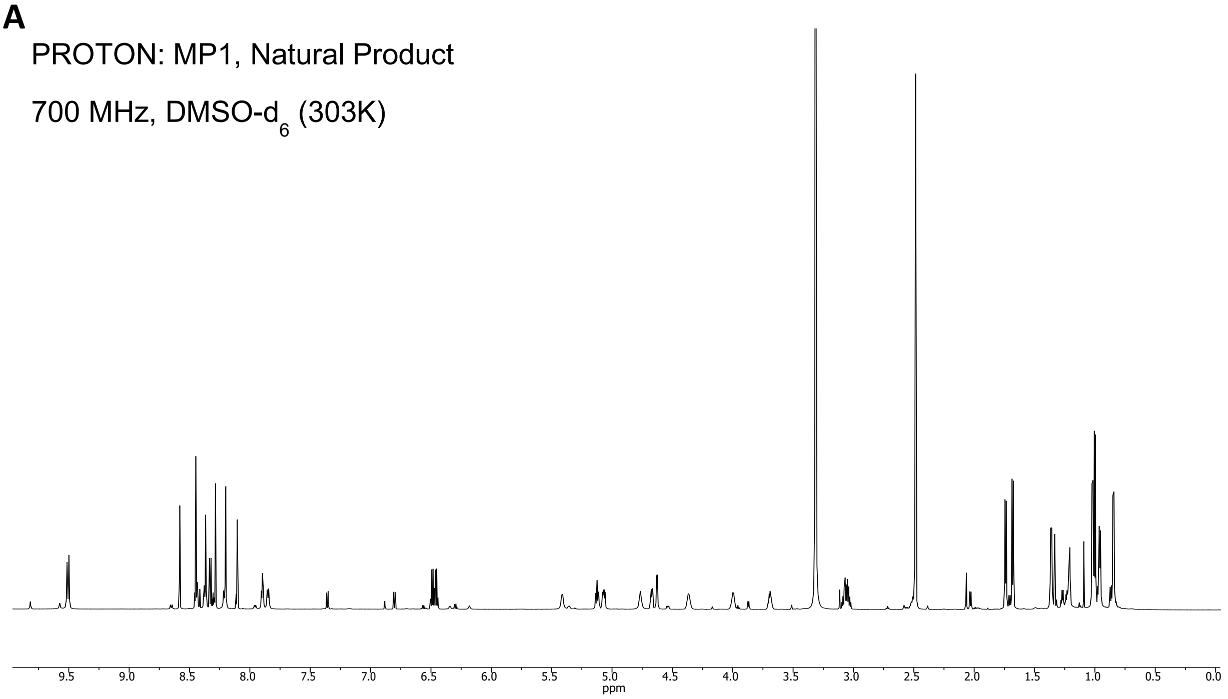

B

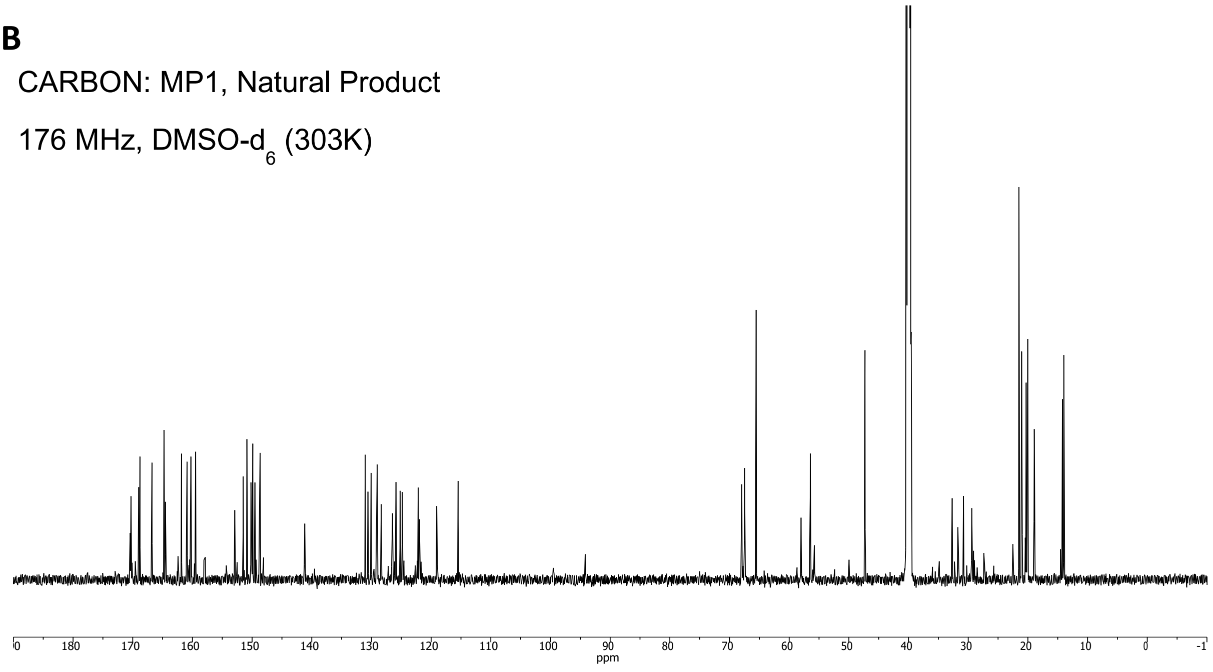

Figure S4: A) ^{1}H NMR spectrum of isolated natural MP1 (10 mg) in DMSO-d_6 (303K). **B)** ^{13}C NMR spectrum of isolated natural M1 (10 mg) in DMSO-d_6 (303K). NMR spectra were recorded on a Bruker AvanceIII-700 (^{1}H: 700 MHz, ^{13}C: 176 MHz).

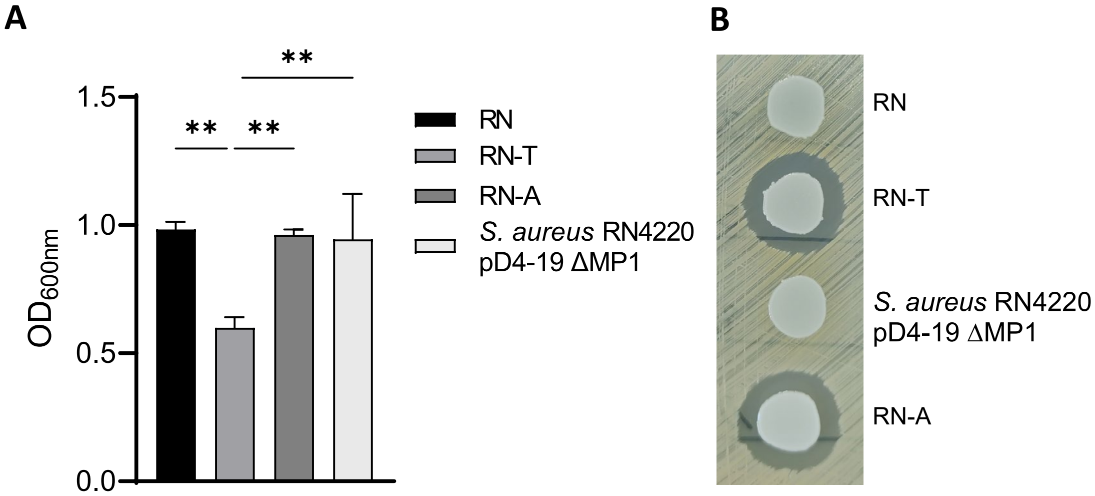

C

	100 µg/ml	50 µg/ml	25 µg/ml	12.5 µg/ml	6.25 µg/ml	3.13 µg/ml	1.56 µg/ml	0.78 µg/ml	0.39 µg/ml	0.2 µg/ml	GC
S. aureus USA 300	0.156	0.099	0.043	0.035	0.025	0.021	0.012	0.019	0.130	-0.001	1.402
S. aureus RN4220	0.121	0.069	0.025	0.010	0.004	0.000	0.008	0.004	0.009	0.006	0.969
S. aureus RN4220 pD4-19	1.243	1.261	1.175	0.928	1.219	1.026	1.020	1.085	1.277	1.257	1.305
S. aureus RN4220 pD4-19 ΔPP	1.326	1.275	1.229	1.267	1.264	1.271	1.240	1.248	1.331	1.260	1.306
S. aureus D4-19	1.385	1.434	1.414	1.433	1.403	1.432	1.418	1.427	1.424	1.428	1.370
S. aureus D4-19 ΔPP	1.341	1.381	1.356	1.354	1.337	1.334	1.354	1.329	1.356	1.343	1.341

Figure S5: A) Endpoint OD_{600nm} of *S. aureus* RN, RN-T, RN-A and *S. aureus* RN4220 pD4-19 ΔMP1 after 20 h in a 24-well microtiter plate (n=3). **B)** Spot assay of *S. aureus* RN, RN-T, RN-A and *S. aureus* RN4220 pD4-19 ΔMP1 on BM plate with *S. aureus* USA300 LAC as indicator strain. Statistical significance was calculated using an ordinary One-way ANOVA (Tukey's multiple comparisons test) (****p < 0.0001). **C)** MIC values of purified MP1 were assessed against *S. aureus* USA300, *S. aureus* RN4220, *S. aureus* RN4220 pD4-19, *S. aureus* RN4220 pD4-19 ΔPP, *S. aureus* D4-19, *S. aureus* D4-19 ΔPP. Mean of three replicates is shown. The highest concentration of MP1 (100 µg/ml) leads to the formation of a precipitate which explains the higher OD values for the two wild type strains at this MP1 concentration. A growth control (GC) is included where no MP1 was added to the well.

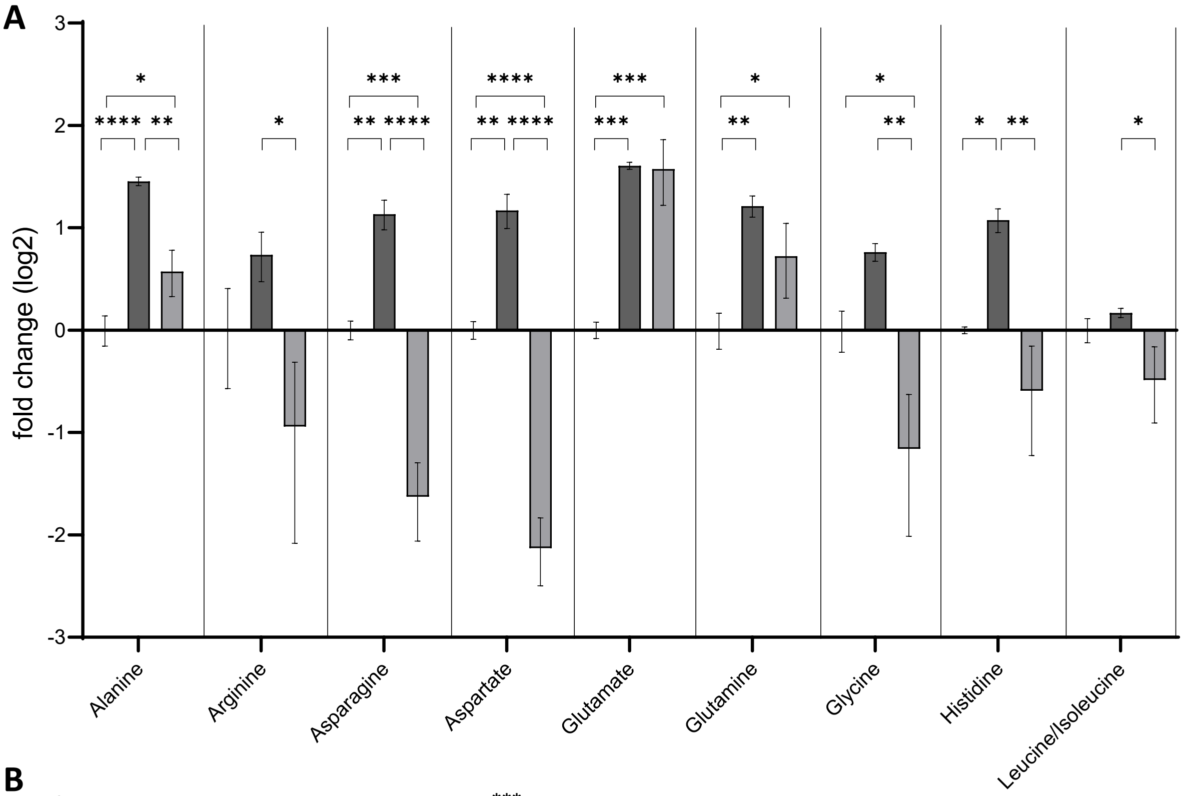

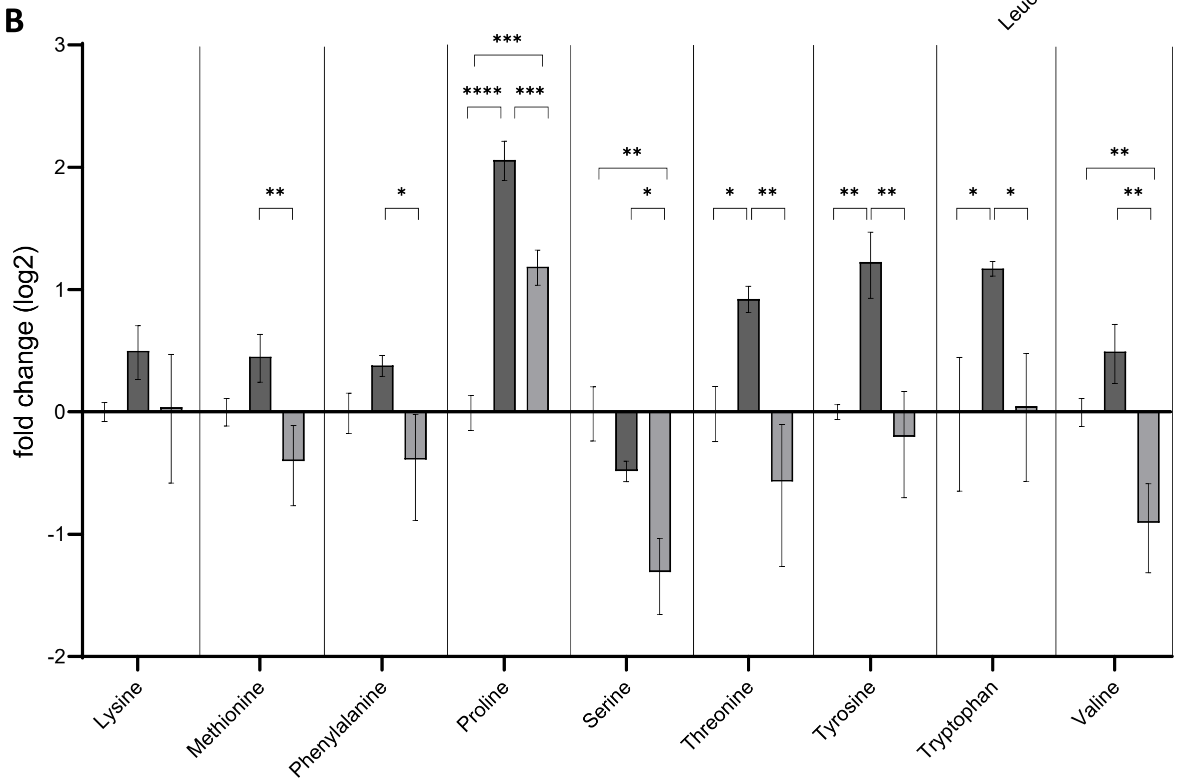

Figure S6: A) Amino acid levels as measured via metabolome analysis in *S. aureus* RN (black), RN-T (dark grey) and RN-A (light grey). Metabolite concentrations are shown as log2-fold changes that are normalized to *S. aureus* RN4220. All amino acids were measured using LC-MS/MS. **B)** Amino acid levels as detected via metabolome analysis in *S. aureus* RN (black), RN-T (dark grey) and RN-A (light grey). Metabolite concentrations are shown as log2-fold changes that are normalized to *S. aureus* RN4220. All amino acids were measured using LC-MS/MS. The p-values were calculated using an ordinary One-way ANOVA (Tukey's multiple comparisons test). Statistical significance is indicated by *p < 0.05, **p < 0.01, ***p < 0.001, ****p < 0.0001.

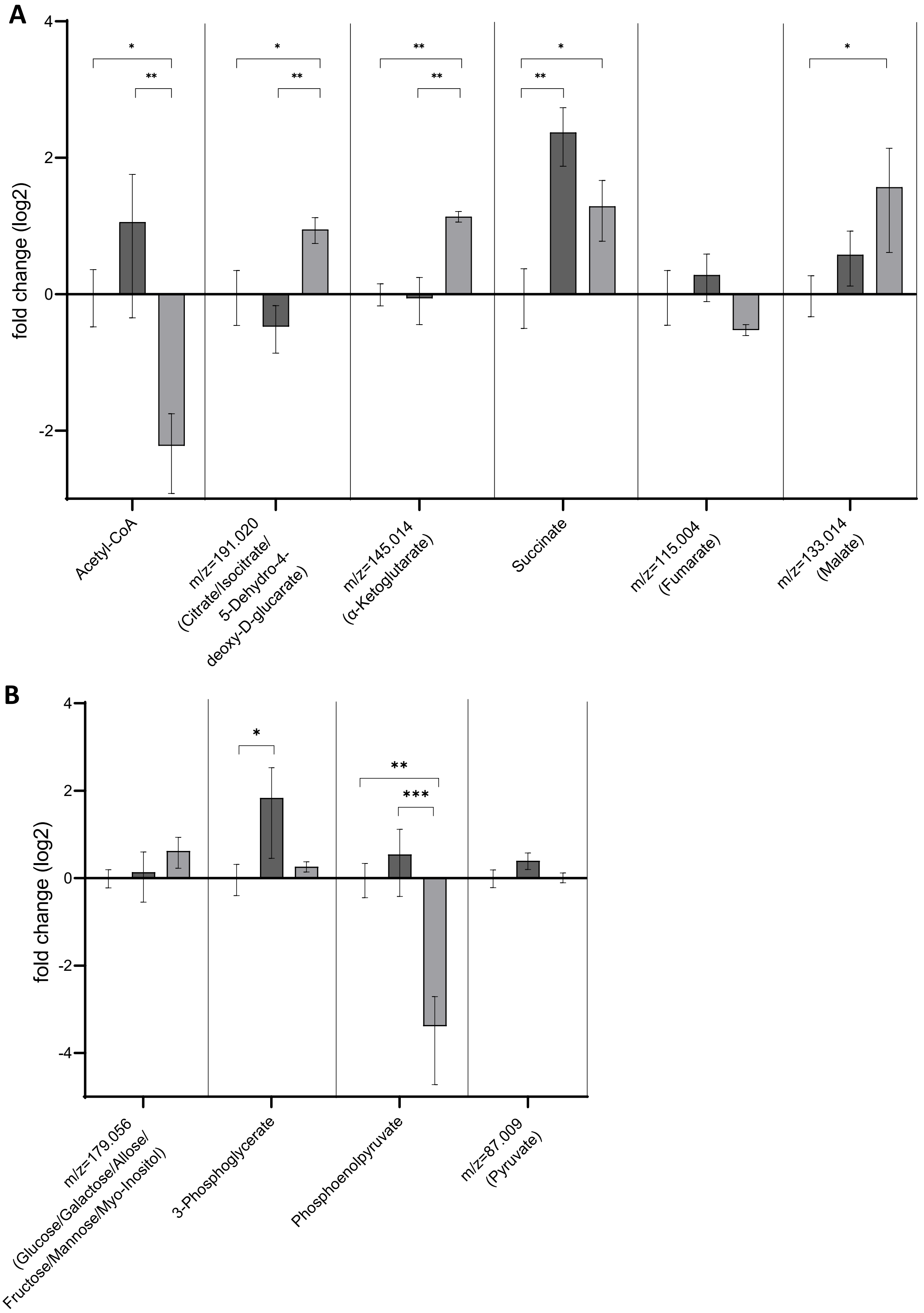
A
4
2
0
-2
fold change (log2)
*
**
*
**
**
**
*
**
*
Acetyl-CoA
m/z=191.020
(Citrate/Isocitrate/
5-Dehydro-4-
deoxy-D-glucarate)
m/z=145.014
(α-Ketoglutarate)
Succinate
m/z=115.004
(Fumarate)
m/z=133.014
(Malate)
B
4
2
0
-2
-4
fold change (log2)
*
**
***
m/z=179.056
(Glucose/Galactose/Allose/
Fructose/Mannose/Myo-Inositol)
3-Phosphoglycerate
Phosphoenolpyruvate
m/z=87.009
(Pyruvate)

Figure S7: A) TCA intermediates as measured via metabolome analysis in *S. aureus* RN (black), RN-T (dark grey) and RN-A (light grey). Metabolite concentrations (acetyl-CoA and succinate) or ion intensities of m/z features (m/z=191.020 matches deprotonated citrate and its isobars isocitrate and 5-dehydro-4-deoxy-D-glucarate, m/z=145.014 matches deprotonated α-ketoglutarate, m/z=115.004 matches deprotonated fumarate and m/z=133.014 matches deprotonated malate) are shown as log2-fold changes that are normalized to *S. aureus* RN4220. **B)** Glycolysis intermediates detected via metabolome analysis in *S. aureus* RN (black), RN-T (dark grey) and RN-A (light grey). Metabolite concentrations (3-phosphoglycerate and phosphoenolpyruvate) or ion intensities of m/z features (m/z=179.056 matches deprotonated glucose and its isobars galactose, allose, fructose, mannose and myo-inositol and m/z=87.009 matches deprotonated pyruvate) are shown as log2-fold changes that are normalized to *S. aureus* RN4220. The p-values were calculated using an ordinary One-way ANOVA (Tukey's multiple comparisons test). Statistical significance is indicated by *$p < 0.05$, **$p < 0.01$, ***$p < 0.001$, ****$p < 0.0001$.

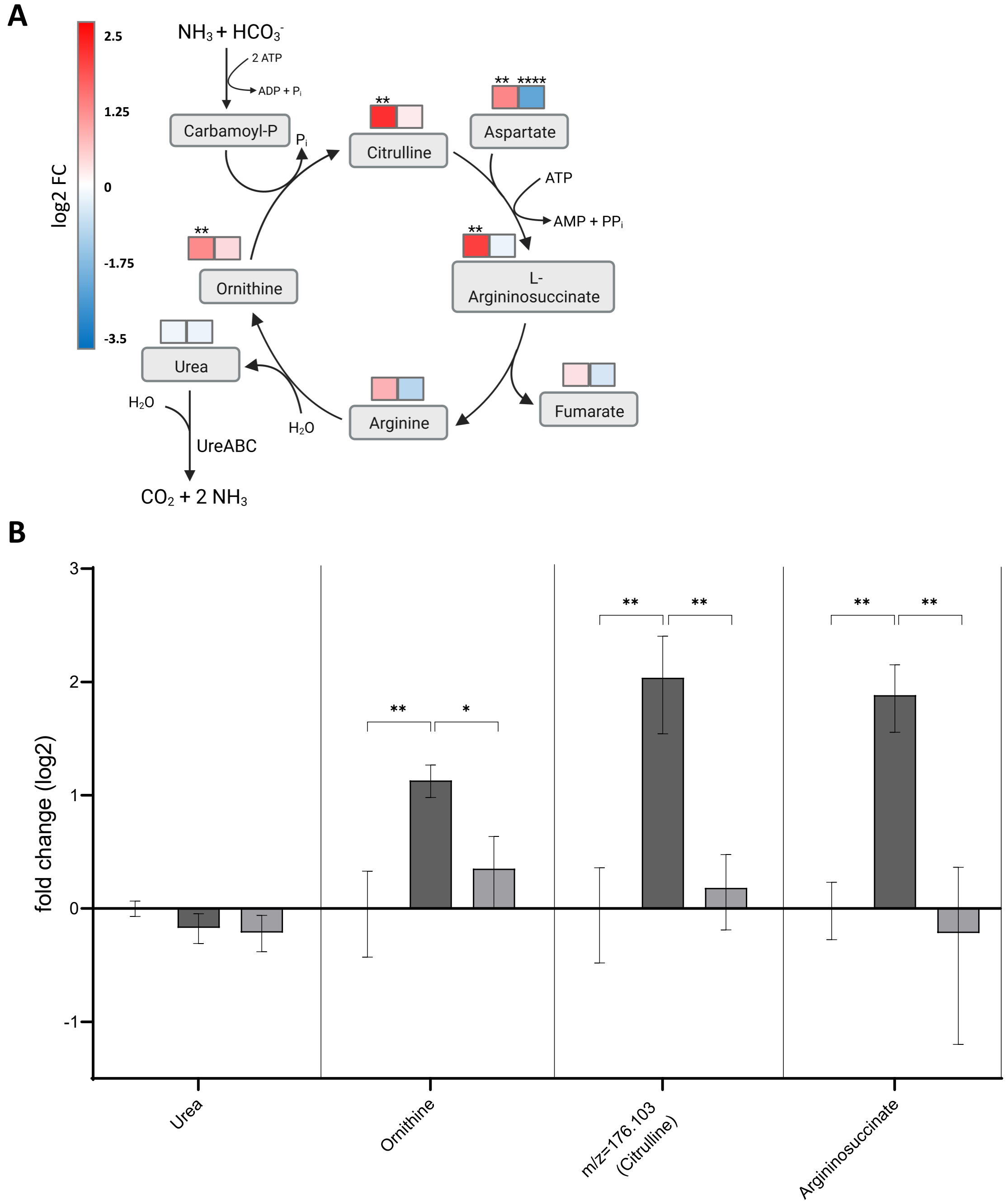

Figure S8: A) Summary of the metabolic differences in urea cycle intermediates between *S. aureus* RN-T and RN-A. The indicated log2 fold changes were calculated for RN-T (left box) and RN-A (right box) vs. *S. aureus* RN4220, respectively. The fold change is indicated by different colours: a decrease in metabolite levels compared to *S. aureus* RN4220 is depicted in blue, an increase in metabolite levels compared to *S. aureus* RN4220 is depicted in red. Created with BioRender.com. **B)** Urea cycle intermediates as measured via metabolome analysis in *S. aureus* RN (black), RN-T (dark grey) and RN-A (light grey). Metabolite concentrations (urea, ornithine, argininosuccinate) or ion intensities of m/z features (m/z=176.103 matches protonated citrulline) are shown as log2-fold changes that are normalized to *S. aureus* RN4220. The p-values were calculated using an ordinary One-way ANOVA (Tukey's multiple comparisons test). Statistical significance is indicated by *p < 0.05, **p < 0.01, ***p < 0.001, ****p < 0.0001.

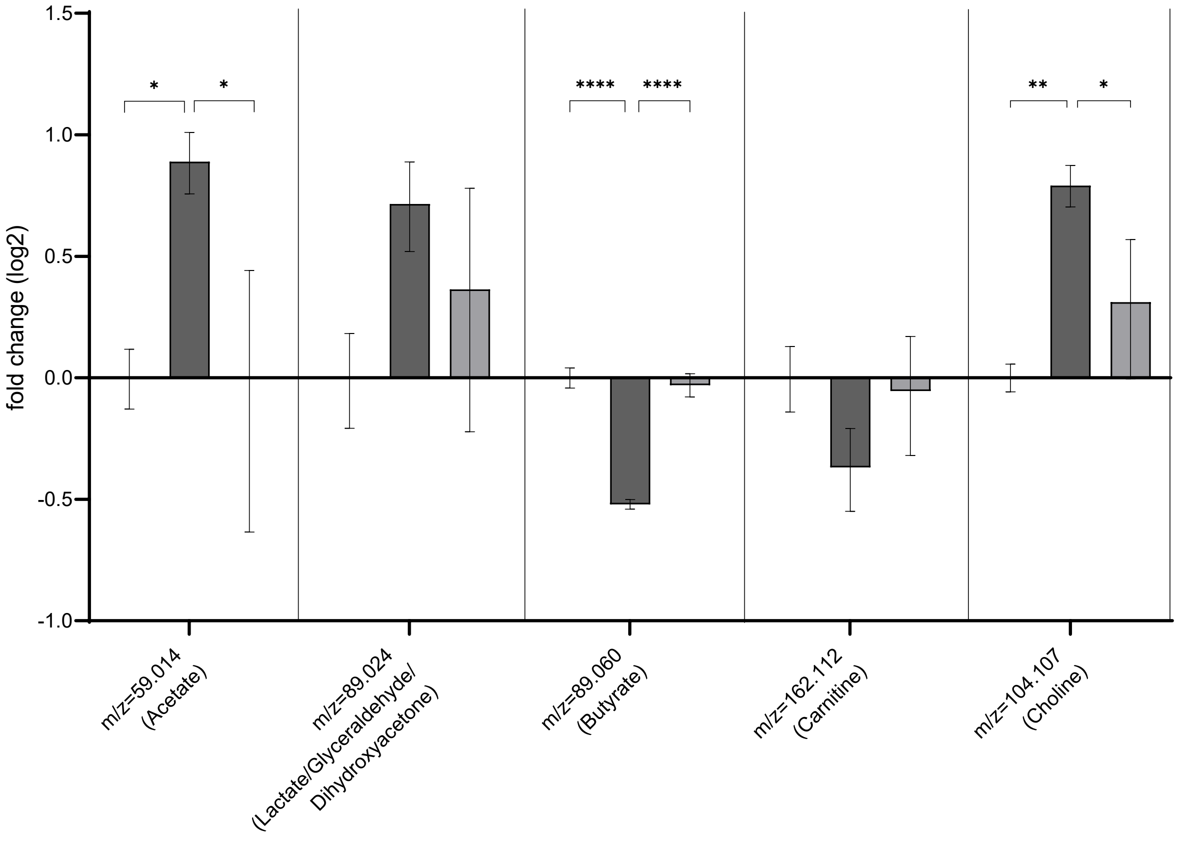

Figure S9: Fermentation products and osmoprotectants as measured via metabolome analysis in *S. aureus* RN (black), RN-T (dark grey) and RN-A (light grey). Ion intensities of m/z features (m/z=59.014 matches deprotonated acetate, m/z=89.024 matches deprotonated lactate and its isobars glyceraldehyde and dihydroxyacetone, m/z=89.060 matches protonated butyrate, m/z=162.112 matches protonated carnitine and m/z=104.107 matches protonated choline) are shown as log2-fold changes that are normalized to *S. aureus* RN4220. The p-values were calculated using an ordinary One-way ANOVA (Tukey's multiple comparisons test). Statistical significance is indicated by *$p < 0.05$, **$p < 0.01$, ***$p < 0.001$, ****$p < 0.0001$.

A

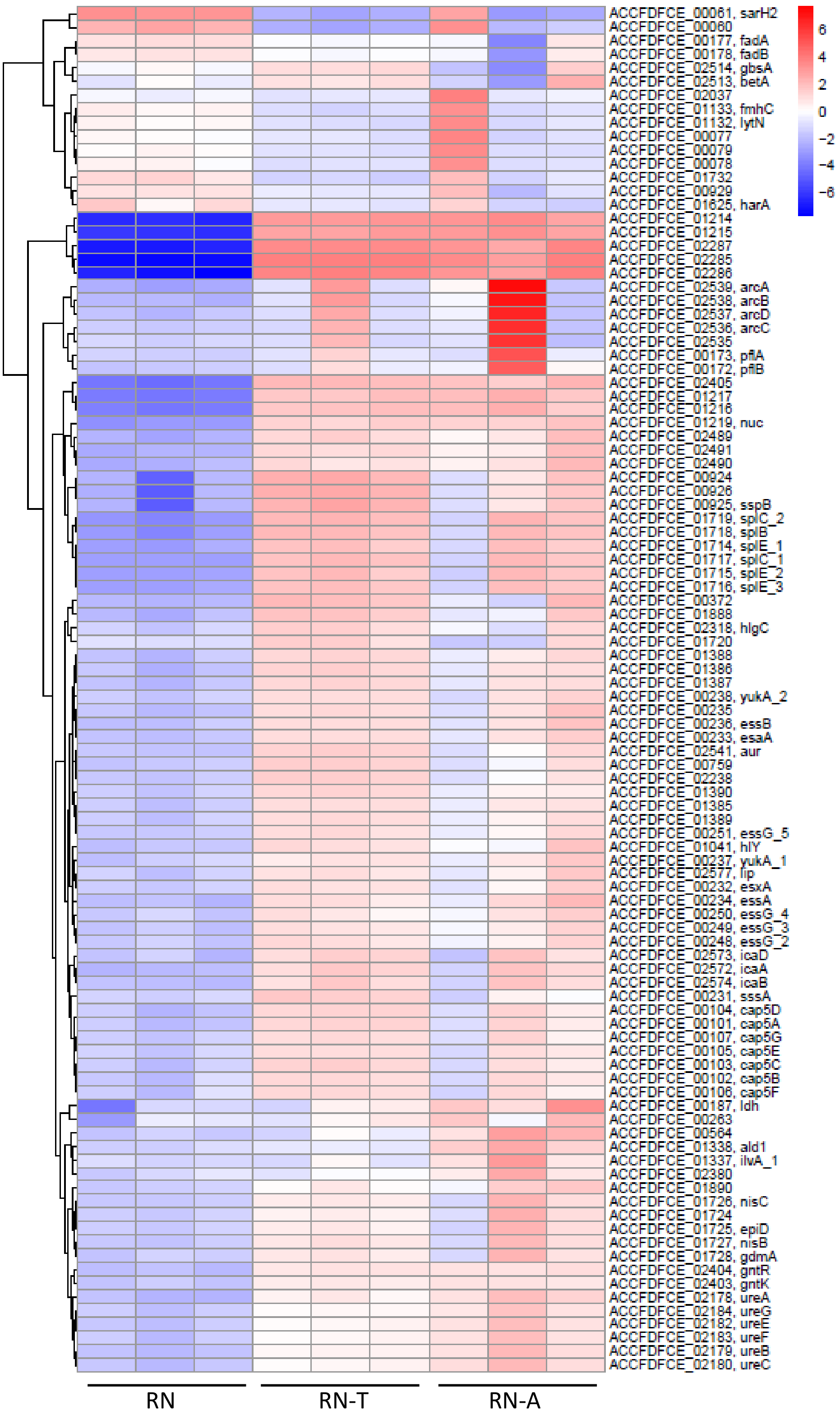

ACCFDFCE_00061, sarH2
ACCFDFCE_00060
ACCFDFCE_00177, fadA
ACCFDFCE_00178, fadB
ACCFDFCE_02514, gbsA
ACCFDFCE_02513, betA
ACCFDFCE_02037
ACCFDFCE_01133, fmhC
ACCFDFCE_01132, lytN
ACCFDFCE_00077
ACCFDFCE_00079
ACCFDFCE_00078
ACCFDFCE_01732
ACCFDFCE_00929
ACCFDFCE_01625, harA
ACCFDFCE_01214
ACCFDFCE_01215
ACCFDFCE_02287
ACCFDFCE_02285
ACCFDFCE_02286
ACCFDFCE_02539, arcA
ACCFDFCE_02538, arcB
ACCFDFCE_02537, arcD
ACCFDFCE_02536, arcC
ACCFDFCE_02535
ACCFDFCE_00173, pflA
ACCFDFCE_00172, pflB
ACCFDFCE_02405
ACCFDFCE_01217
ACCFDFCE_01216
ACCFDFCE_01219, nuc
ACCFDFCE_02489
ACCFDFCE_02491
ACCFDFCE_02490
ACCFDFCE_00924
ACCFDFCE_00926
ACCFDFCE_00925, sspB
ACCFDFCE_01719, splC_2
ACCFDFCE_01718, splB
ACCFDFCE_01714, splE_1
ACCFDFCE_01717, splC_1
ACCFDFCE_01715, splE_2
ACCFDFCE_01716, splE_3
ACCFDFCE_00372
ACCFDFCE_01888
ACCFDFCE_02318, hlgC
ACCFDFCE_01720
ACCFDFCE_01388
ACCFDFCE_01386
ACCFDFCE_01387
ACCFDFCE_00238, yukA_2
ACCFDFCE_00235
ACCFDFCE_00236, essB
ACCFDFCE_00233, esaA
ACCFDFCE_02541, aur
ACCFDFCE_00759
ACCFDFCE_02238
ACCFDFCE_01390
ACCFDFCE_01385
ACCFDFCE_01389
ACCFDFCE_00251, essG_5
ACCFDFCE_01041, hlY
ACCFDFCE_00237, yukA_1
ACCFDFCE_02577, lip
ACCFDFCE_00232, esxA
ACCFDFCE_00234, essA
ACCFDFCE_00250, essG_4
ACCFDFCE_00249, essG_3
ACCFDFCE_00248, essG_2
ACCFDFCE_02573, icaD
ACCFDFCE_02572, icaA
ACCFDFCE_02574, icaB
ACCFDFCE_00231, sssA
ACCFDFCE_00104, cap5D
ACCFDFCE_00101, cap5A
ACCFDFCE_00107, cap5G
ACCFDFCE_00105, cap5E
ACCFDFCE_00103, cap5C
ACCFDFCE_00102, cap5B
ACCFDFCE_00106, cap5F
ACCFDFCE_00187, ldh
ACCFDFCE_00263
ACCFDFCE_00564
ACCFDFCE_01338, ald1
ACCFDFCE_01337, ilvA_1
ACCFDFCE_02380
ACCFDFCE_01890
ACCFDFCE_01726, nisC
ACCFDFCE_01724
ACCFDFCE_01725, epiD
ACCFDFCE_01727, nisB
ACCFDFCE_01728, gdmA
ACCFDFCE_02404, gntR
ACCFDFCE_02403, gntK
ACCFDFCE_02178, ureA
ACCFDFCE_02184, ureG
ACCFDFCE_02182, ureE
ACCFDFCE_02183, ureF
ACCFDFCE_02179, ureB
ACCFDFCE_02180, ureC
6
4
2
0
−2
−4
−6
RN
RN-T
RN-A

B

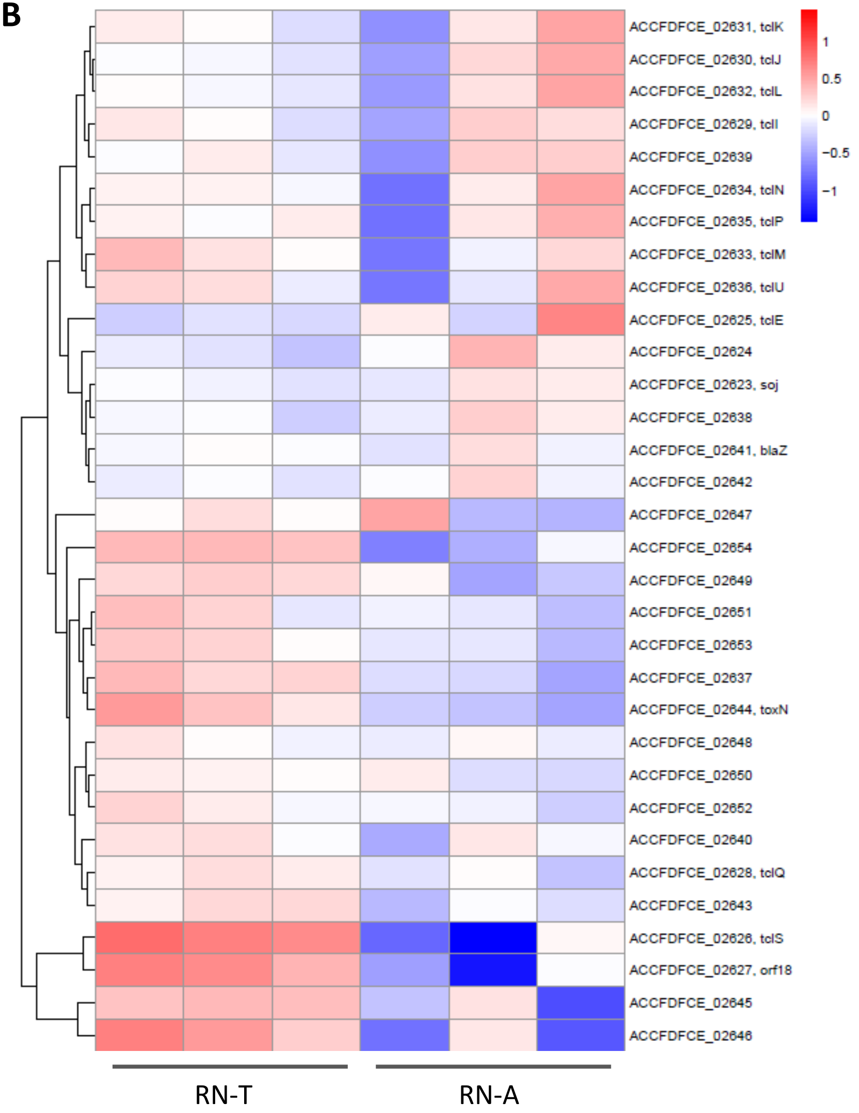

Figure S10: Heat maps showing normalized expression (variance stabilizing transformation) centred on the mean of each row. **A)** Heat map of the 100 most variant genes in *S. aureus* RN, RN-T and RN-A. **B)** Heat map of all genes expressed on pD4-19 in *S. aureus* RN-T and RN-A.
